# Integrated Computational Approaches for Inhibiting Sex Hormone-Binding Globulin in Male Infertility by Screening Potent Phytochemicals

**DOI:** 10.3390/life13020476

**Published:** 2023-02-09

**Authors:** Suvro Biswas, Mohasana Akter Mita, Shamima Afrose, Md. Robiul Hasan, Md. Tarikul Islam, Md. Ashiqur Rahman, Mst. Jasmin Ara, Md. Bakhtiar Abid Chowdhury, Habibatun Naher Meem, Md. Mamunuzzaman, Tanvir Ahammad, Istiaq Uddin Ashik, Munjed M. Ibrahim, Mohammad Tarique Imam, Mohammad Akbar Hossain, Md. Abu Saleh

**Affiliations:** 1 Miocrobiology Laboratory, Department of Genetic Engineering and Biotechnology, University of Rajshahi, Rajshahi 6205, Bangladesh; 2 Department of Genetic Engineering and Biotechnology, University of Rajshahi, Rajshahi 6205, Bangladesh; 3 Department of Pharmaceutical Chemistry, College of Pharmacy, Umm Al-Qura University, Makkah 21955, Saudi Arabia; 4 Department of Clinical Pharmacy, College of Pharmacy, Prince Sattam Bin Abdul Aziz University, Al Kharj, Pin 11942, Saudi Arabia; 5 Department of Pharmacology and Toxicology, Faculty of Medicine in Al-Qunfudah, Umm Al-Qura University, Makkah 28814, Saudi Arabia

**Keywords:** SHBG, male infertility, phytochemicals, molecular docking, ADMET (absorption, distribution, metabolism, excretion, and toxicity), molecular dynamics

## Abstract

Male infertility is significantly influenced by the plasma-protein sex hormone-binding globulin (SHBG). Male infertility, erectile dysfunction, prostate cancer, and several other male reproductive system diseases are all caused by reduced testosterone bioavailability due to its binding to SHBG. In this study, we have identified 345 phytochemicals from 200 literature reviews that potentially inhibit severe acute respiratory syndrome coronavirus 2. Only a few studies have been done using the SARS-CoV-2 inhibitors to identify the SHBG inhibitor, which is thought to be the main protein responsible for male infertility. In virtual-screening and molecular-docking experiments, cryptomisrine, dorsilurin E, and isoiguesterin were identified as potential SHBG inhibitors with binding affinities of −9.2, −9.0, and −8.8 kcal/mol, respectively. They were also found to have higher binding affinities than the control drug anastrozole (−7.0 kcal/mol). In addition to favorable pharmacological properties, these top three phytochemicals showed no adverse effects in pharmacokinetic evaluations. Several molecular dynamics simulation profiles’ root-mean-square deviation, radius of gyration, root-mean-square fluctuation, hydrogen bonds, and solvent-accessible surface area supported the top three protein–ligand complexes’ better firmness and stability than the control drug throughout the 100 ns simulation period. These combinatorial drug-design approaches indicate that these three phytochemicals could be developed as potential drugs to treat male infertility.

## 1. Introduction

Infertility is a reproductive system disease resulting in an inability to achieve a clinical pregnancy despite regular unprotected sexual intercourse for ≥12 months, impacting approximately 72.4 million couples globally [1,2,3]. Among the estimated 8–12% of reproductive-aged couples affected worldwide, 20–30% of infertility cases are exclusively due to male infertility, contributing to 50% of overall cases [4,5,6]. Male infertility impedes spermatogenesis, diminishing the quality and quantity of sperm, and is often observed as altered sperm concentration, motility, and morphology in nearly 7% of all males [1,7,8,9]. Male infertility can be categorized into defective spermatogenesis, defective transport, and ineffective delivery. According to the US Centers for Disease Control and Prevention, about 40–50% of cases are due to male-factor infertility, and 2% are due to suboptimal sperm parameters [1,10]. The reasons underlying male infertility include chronic liver diseases, diabetes mellitus, chronic smoking, insufficient vitamins, coronary heart diseases, and a few genetic factors that adversely affect spermatogenesis [9,11].

An in-depth literature review indicated that infertility could arise due to decreased androgen levels, which play a major role in normal spermatogenesis maintenance. Infertility due to reduced testicular function is also common, the symptoms reflecting reduced testosterone production and serum and intratesticular levels due to reduced gonadotropin (e.g., follicle-stimulating hormone and luteinizing hormone) production regulated at the pituitary level by estrogen [12,13]. Therefore, estrogen has a direct deleterious effect on spermatogenesis since reduced testosterone–estrogen ratios are observed in infertility cases [12,14,15]. Notably, a balance between serum androgens and estrogens is required for normal semen parameters, suggesting a disrupted endocrine mechanism through binding to nuclear receptors, including the estrogen and androgen receptors, because their entry into target cells is essentially regulated by a few serum proteins. Therefore, a crucial transport protein in the serum capable of influencing sex hormone activity, is sex hormone binding globulin (SHBG), which has varying concentrations among individuals. SHBG has been studied extensively. It can bind estrogens and androgens, altering their bioavailability for entry into target cells and tissues since it binds estradiol and testosterone with high affinity, resulting in selective sex-hormone transport in plasma [16,17,18,19].

SHBG is a plasma glycoprotein secreted by the liver that exists as a homodimer comprising two identical monomers, encoded by the 4 kb *SHBG* gene located on the short arm of chromosome 17 (p12–p13 bands) that comprises seven introns and eight exons [20,21,22]. The *SHBG* gene is translated into a 402 amino-acid protein cleaved to release its 29-amino-acid N-terminal sorting peptide. SHBG homodimers have a sex hormone-binding site created by the two monomers, which form a sandwich-like structure capable of binding a single sex hormone, indicating the requirement of SHBG monomer polymerization for the sex hormone-binding site [20,21,22,23]. In the hepatocytes, the SHBG gene’s transcription unit is expressed under the control of a promoter region that is around 800-bp long. The mature SHBG monomer is made up of two laminin G-like (LG) domains and the signal polypeptide that is necessary for secretion, which is encoded by the exons. The translation initiation site for the SHBG-prototype polypeptide sequence, which consists of the signal polypeptide sequence that is terminated during the secretion of the mature polypeptide and the three amino-terminal residues pertaining to the mature SHBG protein, is included in exon 1, which also contains a 60-bp untranslated region. The highly conserved steroid-binding location for vertebrate species is found in the amino-terminal LG domain, which is encoded by exons 2 to 5. A serine residue deep within the binding pocket, like Ser42 in human SHBG, seems to be essential for steroid binding [24,25,26].

The SHBG dimer shows a binding affinity towards sex steroids such as testosterone, dihydrotestosterone, and estradiol, to a lesser extent. It transports sex hormones, regulating their plasma levels and bioavailability for responsive tissues and, overall, demonstrating SHBG’s ability to orchestrate reproductive function and sexual features in males and females [20,21,25,27]. SHBG reduces free testosterone levels, inhibiting the biological induction in reproductive organs by sex hormones and impacting normal reproductive system activity. Testosterone binding to SHBG reduces its bioavailability, preventing it from completing its physiological functions. This disruption causes male infertility, gonadal and erectile dysfunction, prostate cancer, and several male reproductive system diseases with testosterone-dominated male sex-hormone symptoms [21,28]. However, increased bioavailable testosterone levels result in metabolic and reproductive phenotypes. They arise when SHBG’s plasma concentration is decreased, reflecting its role in human metabolism since SHBG concentrations vary in cancer, type 2 diabetes, and dyslipidemia [29,30,31,32]. Additionally, mutations, including single nucleotide polymorphisms, which alter SHBG expression and functions that regulate sperm count and semen quality, are associated with human male infertility [21,33].

Therefore, it can be concluded that the high molecular-weight SHBG plasma protein has a central role in maintaining the balance between bound and unbound sex steroids by attaching to androgens and estrogens with high ligand-binding affinity. SHBG likely alters their access and distribution to their objective tissues through changes in its concentration, identifying SHBG as a therapeutic target for preventing male sterility [25,34]. Besides natural steroid hormones, including testosterone, dihydrotestosterone, and estradiol, SHBG binds numerous endocrine-disrupting chemicals such as phthalates esters, a potential pathway for inhibiting the natural ligand–protein interactions that maintain normal activity in the steroid target organs [34,35,36]. Therefore, natural SHBG inhibitors could be used to treat male infertility.

Virtual screening methods, such as the molecular docking and molecular dynamics (MD) simulation, are reliable high-throughput screening approaches that identify candidate inhibitors from a diverse phytochemical library through their better binding energy and bond stability in simulated molecular interactions with a target protein substrate. In addition, computational assessments of binding modes and bonds are preferred for rapidly identifying phytochemical-like ligand inhibitors for a specific target protein. Therefore, in this study, we aimed to identify effective inhibitors and plausible therapeutic targets to block SHBG function and prevent male sterility by calculating binding affinities and modes and the protein–ligand complex stability between the targeted SHBG protein and numerous phytochemical-based ligands.

## 2. Materials and Methods

### 2.1. Protein Preparation

The SHBG protein’s three-dimensional (3D) structure was retrieved from the RCSB Protein Data Bank (PDB; ID: 1KDM) [37]. Pymol (version 2.5.4) [38] and Discovery Studio (version 4.5.0) [39] were used to dispel and clean the heteroatoms and water molecules from its crystal structure. The Swiss-PDB Viewer (version 4.1) [40] minimized the missing hydrogens, sidechain geometry, improper bond order, and other obligatory factors using the GROMOS (GROningen Molecular Simulation) 43B1 force field.

### 2.2. Ligand Preparation

The ligands were identified from a comprehensive review of 200 articles on severe acute respiratory syndrome coronavirus 2 (SARS-CoV-2), identifying 345 phytochemicals as promising inhibitors. These studies indicated that these 345 phytochemicals were the most potent molecules that successfully inhibited SARS-CoV-2. Following their identification, the PubChem Database [41] was used to collect the 3D structures of these lead phytochemicals and the standard drug, anastrazole. Structure optimization and ligand cleaning, preparation, and minimization were performed using the mmff94 force field [42] with 2000 minimization steps and the perpendicular gradient-optimization algorithm.

### 2.3. Molecular Docking

The PyRx (version 0.9) [43] virtual-screening approach was used to better understand the binding affinity and interaction of candidate ligands with SHBG and standard drug anastrazole with SHBG. Molecular docking was performed in association with the AutoDock Vina protocol. Every potential ligand was converted into PDBQT format to make it suitable for molecular docking, and a universal force field was used to minimize energies. Every bond could be rotated during this process since all of the docking configurations were protein-fixed and ligand-flexible. In AutoDock Vina, a grid box with a center point set of *x* = 2.4963, *y* = 39.1902, and *z* = 29.4898 and the dimensions (in Å) *x* = 44.4875, *y* = 39.2403, and *z* = 41.2118 was formed. The ligand-binding affinity values were shown in negative kcal/mole units, where the best confirmation had the lowest binding-affinity scores. In addition, non-bonding interactions were identified using PyMol, Discovery Studio, and UCSF ChimeraX (version 1.5) [44].

### 2.4. ADMET Prediction

The online servers, pKCSM [45], SwissADME [46], and admetSAR [47] were used to assess the pharmacokinetic properties through ADMET (adsorption, distribution, metabolism, and excretion) predictions. The phytochemicals’ canonical SMILES were retrieved from the PubChem database, and their ADMET properties were estimated by these web servers using SMILES.

### 2.5. MD Simulation

The MD software, YASARA (version 22.9.24) [48,49], and force field, AMBER14 [50,51], were used to perform the MD simulation of the protein–ligand and the protein-standard drug complex. Firstly, the docked complexes were cleaned before their hydrogen-bond network was optimized and oriented. The simulation’s cubic cell with periodic boundary conditions was created using the TIP3P solvation model [52,53,54]. Furthermore, the simulation cell was stretched to 20 Å from the protein–ligand complexes in each direction. The simulation cell’s physiological parameters included 0.9% sodium chloride, a temperature of 298 K, and a pH of 7.4. The steepest gradient algorithm, with 5000 cycles, was used to initially minimize energy in the stimulated annealing system [55,56]. The simulation system’s time step was adjusted to 1.25 femtoseconds (fs). Long-range electrostatic interactions were calculated using the particle-mesh Ewald (PME) system with a cutoff radius of 8.0 Å [57,58,59,60]. Simulation-trajectory data were saved at every 100 picoseconds (ps). Simulations were performed using the Berendsen thermostat, accompanied by fixed pressure and temperature for 100 nanoseconds (ns) [61,62]. The root-mean-square-fluctuation (RMSF), root-mean-square deviation (RMSD), radius of gyration (Rg), solvent-accessible surface area (SASA), and hydrogen bond were examined through the simulation trajectory data [63,64,65,66]. Additionally, using the following equation, the binding free energies of the simulation snapshots were estimated using MM-PBSA (Molecular Mechanics-Poisson Boltzmann Surface Area) techniques.

Binding Energy = E_potRecept_ + E_solvRecept_ + E_potLigand_ + E_solvLigand_ − E_potComplex_ − E_solvComplex_

**Figure 1:**
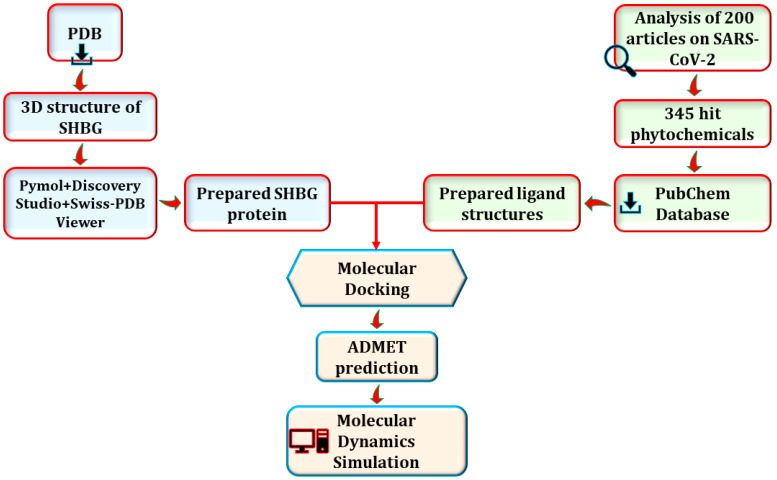
The stepwise procedure of the materials and methods.

The YASARA macro was utilized to calculate the binding free energy for the MM-PBSA system, where a greater positive energy denotes a stronger binding [67,68,69,70,71] affinity. The stepwise procedure in terms of the materials and methods is depicted in Figure 1.

## 3. Results

### 3.1. Molecular Docking

**Figure 2:**
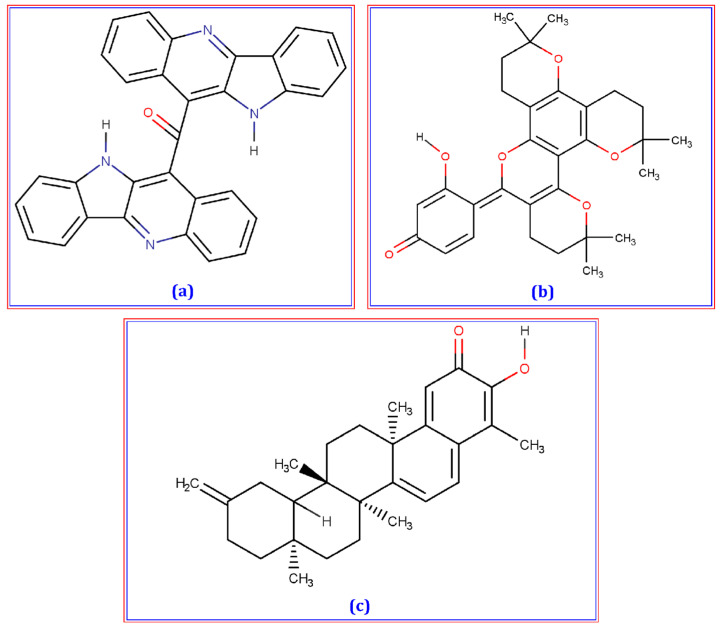
Chemical structure (2D) of Cryptomisrine (**a**), Dorsilurin E (**b**), and Isoiguesterin (**c**). The structures were drawn with the help of Marvin-sketch software.

**Figure 3:**
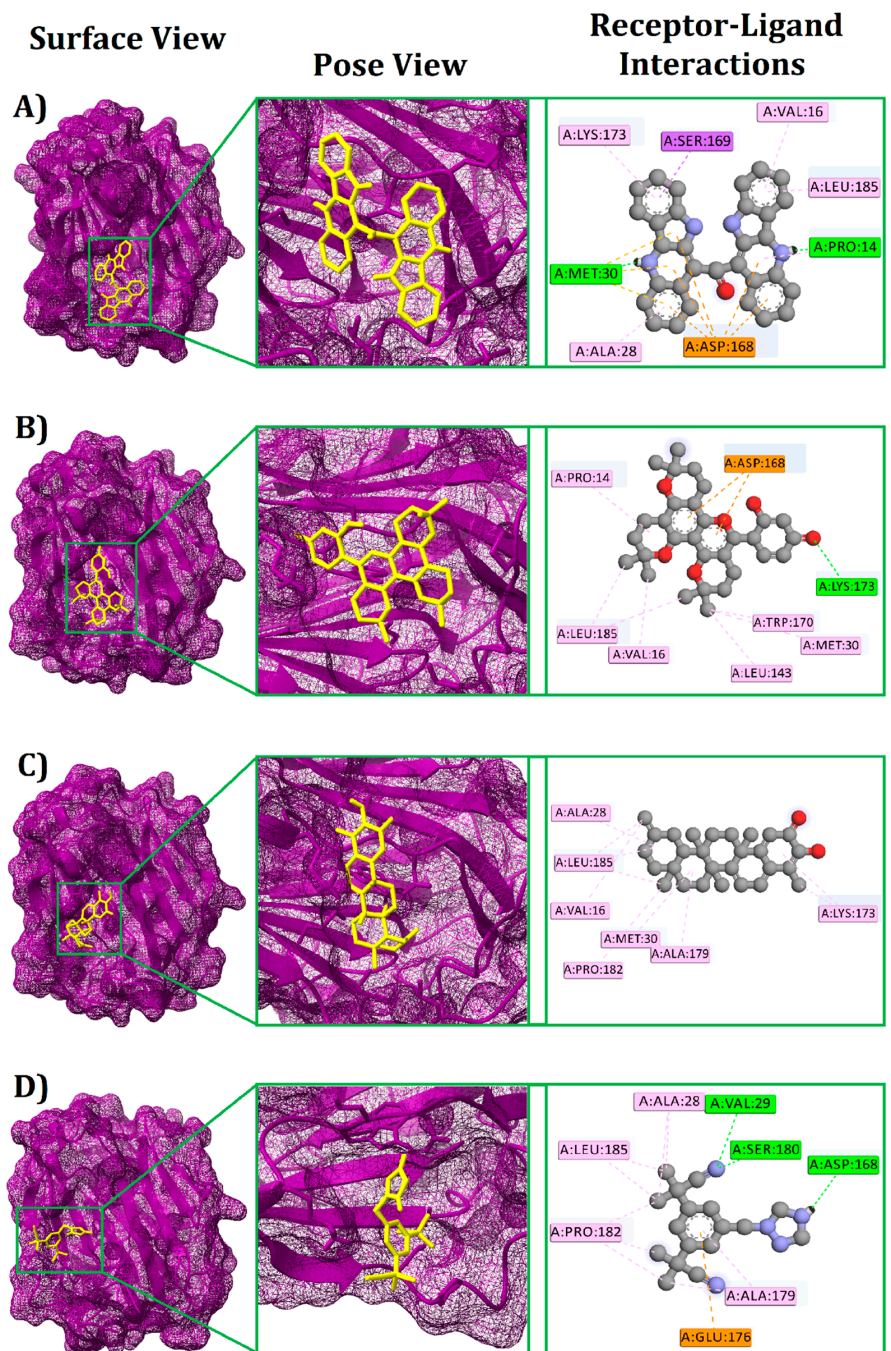
Docking interaction of the SHBG (Sex hormone-binding globulin) protein with the top three hit molecules and the standard drug that shows the surface view, pose view, and receptor–ligand interaction. Here, (**A**) indicates Cryptomisrine, (**B**) indicates Dorsilurin E, (**C**) indicates Isoiguesterin, and (**D**) indicates standard drug (Anastrazole).

The top ten molecules with the highest binding affinity were identified among the 345 candidate phytochemicals (Supplementary File). The three ligands with the best binding affinities (Figure 2) were cryptomisrine, dorsilurin E, and isoiguesterin at −9.2, −9, and −8.8 kcal/mole, respectively (Table 1). The common medication, anastrozole, had a binding affinity of −7.0 kcal/mole to the SHBG protein. Then, PyMol, Discovery Studio, and UCSF ChimeraX were used to look into their non-bond interactions with the SHBG protein. Cryptomisrine formed two conventional hydrogen bonds (at MET30 and PRO14), one electrostatic (Pi-Anion) bond (at ASP168), one hydrophobic (Pi-Sigma) bond (at SER169), and four hydrophobic (Pi-Alkyl) bonds (at LYS173, ALA28, VAL16, and LEU185) with the SHBG protein (Table 2; Figure 3A). Dorsilurin E formed one conventional hydrogen bond (at LYS173), one electrostatic (Pi-Anion) bond (at ASP168), five hydrophobic (Alkyl) bonds (at PRO14, LEU185, VAL16, MET30, and LEU143), and one hydrophobic (Pi-Alkyl) bond (at TRP170) with the SHBG protein (Table 2; Figure 3B). Isoiguesterin formed seven hydrophobic (Alkyl) bonds (at ALA28, MET30, ALA179, LEU185, PRO182, VAL16, and LYS173) with the SHBG protein (Table 2; Figure 3C). Three conventional hydrogen bonds (at VAL29, SER180, and ASP168), one electrostatic (Pi-Anion) bond (at GLU176), and four hydrophobic (Alkyl) bonds (at ALA28, ALA179, PRO182, and LEU185) stabilized the anastrozole-SHBG complex (Table 2; Figure 3D).

### 3.2. ADMET Prediction

The pharmacokinetics and toxicity properties of the three top ligands were evaluated to ensure their efficiency and safety. The molecular weights of cryptomisrine, dorsilurin E, and isoiguesterin were 462.5, 490.6, and 404.6 g/mol, respectively (Table 3). Moreover, they followed Lipinski’s rule of five, which stipulates that a potent molecule should have a molecular weight of ≤500 g/mol [72,73]. Moreover, cryptomisrine, dorsilurin E, and isoiguesterin had 3, 6, and 2 hydrogen-bond acceptors, respectively, and 2, 1, and 1 hydrogen-bond donors, respectively. The topological polar surface areas (TPSAs) of cryptomisrine, dorsilurin E, and isoiguesterin were 74.43, 74.22, and 37.30 Å^2^, respectively. Cryptomisrine, dorsilurin E, and isoiguesterin were 96.507%, 93.133%, and 95.798% absorbed in the human intestinal tract, respectively. In addition, they had no Ames toxicity, skin sensitization, or P-glycoprotein substrates. Cryptomisrine, Dorsilurin E, and Isoiguesterin, each had CNS permeability scores of −1.073, −2.703, and −1.955, respectively. Each of the top three compounds passed the carcinogenicity test and was found to be non-carcinogenic. None of the top three compounds displayed toxicity in the instance of acute oral poisoning. Isoiguesterin, Dorsilurin E, and Cryptomisrine all had BBB permeability ratings of −0.202, −0.368, and −0.7629, respectively. Furthermore, none of the top three compounds exhibited any toxicity during the hepatotoxicity test. Moreover, they followed Lipinski’s rule of five, with one violation for cryptomisrine and isoiguesterin but none for dorsilurin E. However, one lone violation of this rule does not disqualify a candidate from consideration as a viable therapeutic candidate [46].

### 3.3. MD Simulation

**Figure 4:**
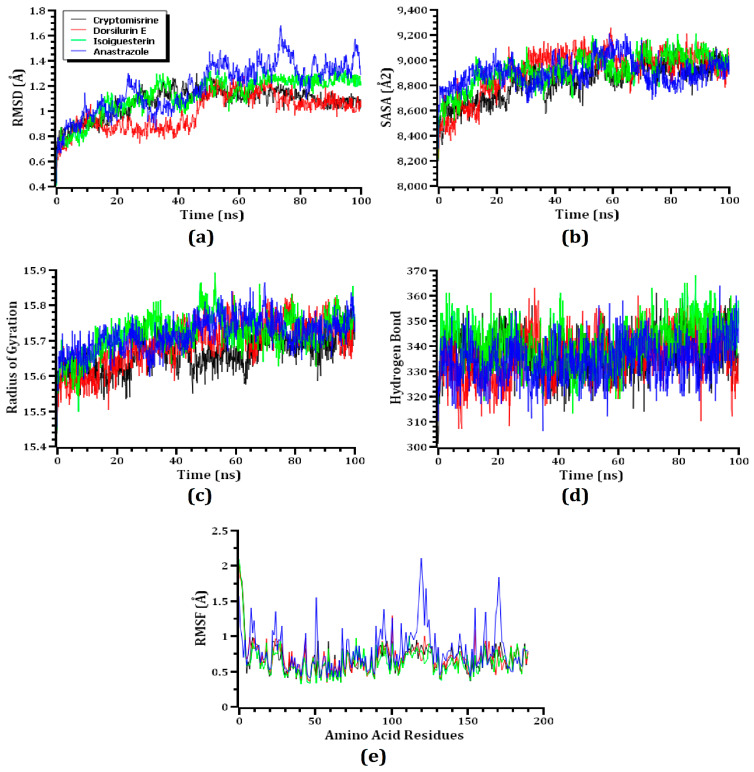
Simulations are analyzed using time-series analyses. As listed in alphabetical order from (**a**–**e**), the RMSD of alpha-carbon atoms is represented by (**a**), protein volume with expansion is represented by (**b**), rigidity and compactness analyses are represented by (**c**), hydrogen bonding of the complexes is represented by (**d**), and the flexibility of amino-acid residue is represented by (**e**).

The top three ligand–protein complexes as well as the standard drug–protein complex were subjected to 100 ns MD simulations to explore their structural firmness and confirm their docking scenarios. The stability of protein–ligand complexes was evaluated by measuring the RMSD of C-alpha atoms. First, the cryptomisrine, dorsilurin E, and isoiguesterin–SHBG complexes’ RMSD increased for the first few seconds of the simulation, indicating their preliminarily higher instability. The RMSD of the isoiguesterin–SHBG complex was generally greater than those of the dorsilurin E–SHBG and cryptomisrine–SHBG complexes (Figure 4a). The dorsilurin E–SHBG showed a lower average RMSD than that of the isoiguesterin–SHBG and cryptomisrine–SHBG complexes. While the RMSD of the dorsilurin E–SHBG complex suddenly increased after 45 ns, it stabilized at around 80 ns and remained stable for the final 20 ns. The RMSD of the isoiguesterin–SHBG complex decreased appreciably after 40 ns but stabilized at around 65 ns and remained stable for the remaining simulation time with only minor fluctuations. The RMSD of the cryptomisrine–SHBG complex fluctuated until it stabilized at 85 ns. RMSD instability was present in the complex containing the reference drug anastrozole throughout the simulation period, with the peak occurring at 75 ns. Nevertheless, all three complexes remained stable throughout the simulation since their RMSD remained <2.5 Å [64].

To examine how the SHBG’s surface changes in response to ligands, SASAs were determined for the three top complexes since this parameter is crucial for understanding protein stability and folding [74]. Higher SASAs imply an enlarged protein surface area, while lower SASAs imply a reduced protein surface area [60]. Between 30–50 ns, the SASAs of the dorsilurin E–SHBG complex were greater than those of the cryptomisrine–SHBG and isoiguesterin–SHBG complexes, indicating that it had a greater surface area (Figure 4b). In addition, the cryptomisrine–SHBG complex had the lowest average of SASAs among these complexes, indicating that it had the smallest surface area. While the cryptomisrine–SHBG, dorsilurin E–SHBG, and isoiguesterin–SHBG complexes showed fluctuating SASAs until 70 ns, they remained relatively stable over the final 30 ns, indicating that they were stable. The SASA value for the complex containing anastrozole initially increased, but after 25 ns of simulation, the value drastically decreased. This complex showed the lowest SASA value of all the complexes after 70 ns, which indicates that the complex’s protein had been truncated.

The protein–ligand complexes were determined as either more rigid or labile by measuring their Rg values. Lower Rg values indicate a more rigid protein–ligand complex, and higher Rg values indicate a more labile protein–ligand complex [56]. All three complexes showed an initial increase in their Rg value. The isoiguesterin–SHBG complex had higher Rg values on average, indicating that it had a more labile nature than the other two complexes during the simulation (Figure 4c). In contrast, the cryptomisrine–SHBG complex had the lowest Rg value between 40 and 65 ns, indicating rigidness. In addition, all three complexes showed very slight fluctuations in Rg values after the initial increase and comparatively lower Rg values, confirming their rigidness throughout the simulation. Compared to the other three complexes, the complex containing the common medicine, anastrazole, had the highest average Rg value across the simulation period, indicating that it was a more labile compound.

The complexes’ hydrogen bonds were evaluated since they play a vital role in sustaining the integrity and stability of the docked complexes during the simulation [75]. The complexes of cryptomisrine–SHBG, dorsilurin E–SHBG, isoiguesterin–SHBG, and anastrozole–SHBG produced a significant number of hydrogen bonds, indicating a robust and rigid complex throughout the simulation (Figure 4d). To better understand SHBG’s suppleness across the amino-acid residues, RMSFs were examined for these three protein–ligand complexes. The RMSFs of all amino acids in the top three protein–ligand complexes did not exceed 2.5 Å. While the first few amino acids initially showed higher RMSFs, they dropped drastically after a few ns (Figure 4e). In general, lower RMSFs correspond to a higher rigidness. It is evident from the lower RMSFs of the top three complexes that they remained stable throughout the simulation [75]. With the largest peak occurring at 120 amino acid residues, the RMSF value for the complex containing anastrozole was larger on average than that of the other three complexes. It is evident from the top three complexes’ lower RMSF values that they remained steady longer than the complex with the typical medicine, anastrazole, throughout the simulation time. Lower RMSF values frequently imply a higher level of firmness.

**Figure 5:**
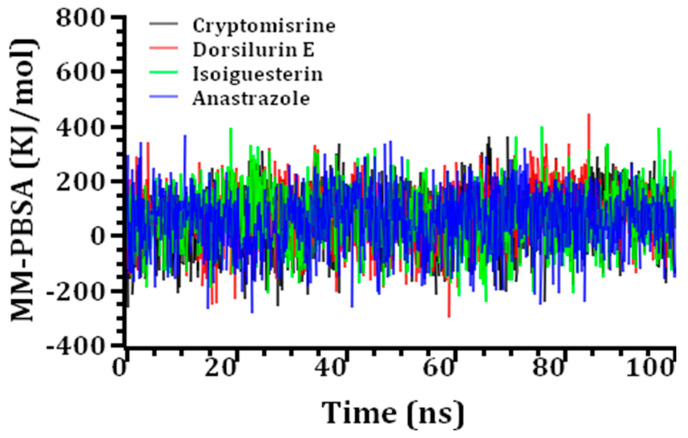
MM-PBSA binding free energy calculations of the top three screened molecules and the standard complex.

A stronger binding is denoted by a greater positive energy, which is shown in Figure 5, along with the results of the MM-PBSA binding free-energy calculation for the top three docked complexes and the common medication, anastrazole. I soiguesterin, dorsilurin E, cryptomisrine, and anastrazole (standard drug) had average binding free energies of 67.64, 71.39, 69.13, and 57.38 KJ/mol, respectively (Table 4). The possible three ligand molecules bind the SHBG protein more effectively than the conventional complex due to their larger average-binding free energies.

## 4. Discussion

While numerous studies have used in silico analyses of distinct disease-causing receptor proteins to predict their bioactive molecules and inhibitors, few have examined the role of potent natural inhibitors of the SHBG protein to prevent male infertility [9,76]. The multifunctional SHBG protein, also known as testosterone-estradiol-binding globulin, is amalgamated by hepatocytes [21]. SHBG transports androgens, estrogens, testosterone, and estradiol in the blood and regulates their entry into target tissues [25,77,78]. Sex hormone levels are particularly altered when SHBG binds sex hormones, affecting their bioavailability. Since testosterone is the dominant male sex hormone, SHBG’s binding to it limits its biological action in the male reproductive system, causing reproductive problems [21]. These changes influence normal male reproductive system activity, leading to reproductive system diseases, such as male infertility (mostly by affecting semen quality and sperm count [79]), sexual dysfunction, erectile dysfunction, and prostate cancer [21]. Plasma SHBG levels and sperm count are lower in infertile men [12]. Therefore, natural inhibitors of the SHBG protein could be used to treat male infertility [76].

A similar computational study of the SHBG protein used molecular docking to examine 47 natural phytochemicals. The most potent natural compound was chlorogenic acid, which had a docking score of −7.255 kcal/mol but was only stable at 10 ns in MD simulations [76]. However, in this study, the three compounds identified (crytomisrine, dorsilurin E, and isoiguesterin) had docking scores of −9.2, −9, and −8.8 kcal/mol, respectively, and were stable at 100 ns in MD simulations. Similarly, Ishfaq et al. examined the binding of nine phthalates with SHBG inhibiting activity (BBP, DNHP, DEHP, DMP, DNOP, DINP, DIDP DBP, and DIBP) to human SHBG, finding docking scores between −6 and −10.12 kcal/mol. However, they did not examine protein–ligand complex stability [34]. In addition, another study explored molecular interactions between human-SHBG and chlorpyrifos and its degradation derivatives, including TMP, TCP, CPYO, and DEC. While they had docking scores between −6.097 and −7.662 kcal/mol, protein–ligand complex stability was not assessed through MD simulations [79]. Therefore, this study’s three selected compounds have more stable molecular interactions and more reasonable binding affinity than previously explored inhibitors.

An in silico analysis of cryptomisrine, an alkaloid extracted from *Cryptolepis sanguinolenta*, found it to be a potent inhibitor of RNA-dependent RNA polymerase and SARS-CoV-2′s main protease (Mpro). It had docking scores of −9.80 and −10.60, respectively, the highest among 13 natural compounds from the plant with molecular-interaction stability during the MD simulation [80]. When docked against two different conformations of the Mpro protein of SARS-CoV 2, including typical substrate binding sites and ligand-induced substrate-binding sites, dorsilurin E had binding energies of −9.51 kcal/mol and −11.31 kcal/mol, respectively [81]. One of the top two terpenoids, with high binding scores, that was isolated from African medicinal plants, isoiguesterin, had −9.5 Kcal/mol binding energies when it interacted with the ACE2 and TMPRSS2 proteins to block the SARS-CoV 2 host-cell entry [82]. Therefore, the chosen molecule has previously shown inhibitory effects against viral proteins, encouraging further analysis against other protein targets for inhibition.

This study has shown that crytomisrine, dorsilurin E, and isoiguesterin strongly bind to the SHBG protein, and their complexes remain stable and rigid during simulations. Their pharmacokinetics and toxicity properties indicate that all three natural compounds are safe and follow Lipinski’s rule for drug candidates. Anastrazole, a medication used to treat male infertility on a regular basis, had a binding affinity calculated at −7.0 kcal/mole, which was lower than the three top compounds in our analysis. Additionally, for the entire molecular dynamic-simulation period, the control medication revealed a more unstable and labile interaction with the target protein than our lead compounds. Additionally, compared to the standard medication, anastrazole, the three lead compounds showed a much-improved RMSD, Rg, RMSF, and SASA profiles. In addition, each of the top three compounds in our investigation had a lower average binding free energy than the common medication, anastrazole. Therefore, we can conclude that these three compounds, with safe ADMET predictions, can be used as drugs for male infertility by inhibiting SHBG-protein activity.

## 5. Conclusions

Phytochemicals are becoming increasingly feasible and more promising therapeutic sources than synthetic constituents due to their broad appearance, wide specificity, and lower side effects. This study integrated many computational approaches to identify effective SHBG inhibitors. A detailed study of 200 articles identified 345 potent phytochemicals with activity against SARS-CoV-2 for screening. Three prospective SHBG inhibitors were cryptomisrine, dorsilurin E, and isoiguesterin, based on virtual screening and molecular docking. These were observed to have higher binding affinities and average binding free energies than the reference drug, anastrazole, and their pharmacokinetic properties satisfied the requirements for promising therapeutic candidates. Additionally, MD simulations verified that the top three docked protein–ligand complexes were more robust and stable than the control medication, anastrazole. Further in vitro experiments are required to establish the precise efficiency of these three drug candidates against SHBG since this combinatorial screening study was exclusively computational.

## Figures and Tables

**Table 1:** The binding affinity of the top 10 lead molecules derived from molecular docking.

Compound Name	Pubchem CID	Docking Score (kcal/mole)
Cryptomisrine	10600127	−9.2
Dorsilurin E	15478906	−9
Isoiguesterin	11373102	−8.8
(-)-Catechin gallate	6419835	−8.6
2,3-dihydroamentoflavone	16066857	−8.4
Epicatechin 3,5-di-O-gallate	14284594	−8.4
Theaflavine	135403798	−8.2
Fortunellin	5317385	−8.1
Cassigarol G	10005549	−8.1
Pseudojervine	16398499	−8

**Table 2:** Docking interactions of the SHBG protein with the top three ligand molecules and the standard drug anastrazole. Data for the interactions were retrieved from the Discovery Studio.

Compound Name	Pubchem CID	Docking Score	Residues in Contact	Interaction Type	Distance in Å
Cryptomisrine	10600127	−9.2	MET30	Conventional Hydrogen Bond	2.8018
			PRO14	Conventional Hydrogen Bond	1.78914
			ASP168	Electrostatic (Pi-Anion)	4.64313
			SER169	Hydrophobic (Pi-Sigma)	2.88827
			LYS173	Hydrophobic (Pi-Alkyl)	4.70796
			ALA28	Hydrophobic (Pi-Alkyl)	5.006
			VAL16	Hydrophobic (Pi-Alkyl)	4.3933
			LEU185	Hydrophobic (Pi-Alkyl)	4.90543
Dorsilurin E	15478906	−9	LYS173	Conventional Hydrogen Bond	2.71543
			ASP168	Electrostatic (Pi-Anion)	3.87237
			PRO14	Hydrophobic (Alkyl)	5.24634
			LEU185	Hydrophobic (Alkyl)	4.233
			VAL16	Hydrophobic (Alkyl)	4.08529
			MET30	Hydrophobic (Alkyl)	5.21275
			LEU143	Hydrophobic (Alkyl)	4.93558
			TRP170	Hydrophobic (Pi-Alkyl)	5.14218
Isoiguesterin	11373102	−8.8	ALA28	Hydrophobic (Alkyl)	4.55253
			MET30	Hydrophobic (Alkyl)	5.09204
			ALA179	Hydrophobic (Alkyl)	4.91895
			LEU185	Hydrophobic (Alkyl)	4.57361
			PRO182	Hydrophobic (Alkyl)	4.51348
			VAL16	Hydrophobic (Alkyl)	4.71484
			LYS173	Hydrophobic (Alkyl)	3.86922
Anastrazole	2187	−7.0	VAL29	Conventional Hydrogen Bond	2.93091
			SER180	Conventional Hydrogen Bond	2.89776
			ASP168	Conventional Hydrogen Bond	2.82215
			GLU176	Electrostatic (Pi-Anion)	4.86148
			ALA28	Hydrophobic (Alkyl)	4.35855
			ALA179	Hydrophobic (Alkyl)	4.14398
			PRO182	Hydrophobic (Alkyl)	4.48299
			LEU185	Hydrophobic (Alkyl)	4.43503

**Table 3:** Analysis of the pharmacological properties of the top three ligand molecules.

Parameters	Cryptomisrine	Dorsilurin E	Isoiguesterin
Molecular weight	462.5 g/mol	490.6 g/mol	404.6 g/mol
Num. H-bond acceptors	3	6	2
Num. H-bond donors	2	1	1
TPSA (S)	74.43 Å^2^	74.22 Å^2^	37.30 Å^2^
AMES toxicity	No	No	No
Human intestinal absorption	96.507 (% Absorbed)	93.133 (% Absorbed)	95.798 (% Absorbed)
Skin Sensitization	No	No	No
P-glycoprotein substrate	No	No	No
CNS permeability	−1.073	−2.703	−1.955
Carcinogenicity	Non-carcinogenic	Non-carcinogenic	Non-carcinogenic
Acute oral toxicity	No	No	No
BBB permeability	0.7629	−0.368	−0.202
Hepatotoxicity	No	No	No
Lipinski rule of five	Yes; 1 violation	Yes; 0 violation	Yes; 1 violation

**Table 4:** MM-PBSA binding free energy calculation of the top three screened molecules and the standard complex Anastrazole.

Compound Name	Average Binding Free Energy (KJ/mol)
Cryptomisrine	67.64
Dorsilurin E	71.39
Isoiguesterin	69.13
Anastrazole	57.38

## Data Availability

All data generated or analyzed during this study are included in this published article.

## Supplementary Materials

The following supporting information can be downloaded at: https://www.mdpi.com/article/10.3390/life13020476/s1.
