# Supplementary material for: Integrated Computational Approaches for Inhibiting Sex Hormone-Binding Globulin in Male Infertility by Screening Potent Phytochemicals
Source: Life (Basel). 2023 Feb 9;13(2):476. doi: 10.3390/life13020476 (PMC9966787; doi:10.3390/life13020476)
Supplement: Supplementary file 1 [file life-13-00476-s001.zip › life-2087967-supplementary.pdf]

Article No.	Compound Name	PubChem CID	References
01	Apigenin	5280443	(Khan et al., 2021)
	kaempferol	5280863	
02	mukonicine	86242003	(Wadanambi et al., 2022)
	o-methylmurrayamine A	14892681	
	koenine	5318827	
	girinimbine	96943	
03	Dehydroglyasperin C	480775	(Hejazi et al., 2021)
	Licochalcone D	10473311	
	Liquiritin	503737	
04	Luteolin	5280445	(Ferdous et al., 2021)
	myricetin	5281672	
	quercetin	5280343	
05	alterporriol-Q	57332381	(Das et al., 2022)
06	epicatechin-3,5-di-O-gallate	14284594	(Bhardwaj et al., 2021)
	epigallocatechin-3,5-di-O-gallate	467299	
	epigallocatechin-3,4-di-O-gallate	467301	
07	corilagin	73568	(Haddad et al., 2022)
	1,3,6-tri-O-galloyl-beta-D-glucose	452707	
08	khainaoside C	44606239	(Adem et al., 2021a)
	6-O-Caffeoylarbutin	15689808	
09	Naringoside	442428	(Faisal et al., 2022)
	Myricetin	5281672	
	Aureusidin 4,6-diglucoside	42607768	
10	linarin	5317025	(Muthumanickam et al., 2021)
	cadinene	3032853	

	geranyl acetate	1549026	
	alpha-Thujene	17868	
	Baicalin	64982	
11	luteolin-7-O-glucuronide	13607752	(Mohapatra et al., 2020)
	chlorogenic acid	1794427	
12	darunavir	213039	(Swain et al., 2022)
	quercetin-3-rhamnoside	5280459	
13	Isorhoifolin	9851181	(Yañez et al., 2021a)
14	curcumin	969516	(Pluskota-Karwatka et al., 2021)
	cannabidiol	644019	
15	Amentoflavone	5281600	(Hossain et al., 2021)
16	moromycin A	25112053	(Al-Bustany et al., 2022)
17	Withanoside V	10700345	(Tripathi et al., 2020a)
18	mangiferin	5281647	(In et al.)
	glucogallin	124375	
	phlorizin	6072	
19	catechin gallate	6419835	(Mahmud et al., 2021a)
	epicatechin-3-O-gallate	107905	
	psi-taraxasterol	5270605	
20	thalimonine	10893946	(Garg and Roy, 2020)
	sophaline D	132991317	
21	Theaflavin	135403798	(Bhardwaj et al., 2022)
	catechin	9064	
22	Salvianolic acid A	5281793	(Ibrahim et al., 2020a)

23	Genistein	5280961	(Manjunathan et al., 2022)
	Quercetin	5280343	
24	chebulinic acid	72284	(Rudrapal et al., 2022)
	terflavin A	16175788	
	corilagin	73568	
	chebulagic acid	250397	
25	licorice glycoside E	42607811	(Lu et al., 2022)
26	4′′′′′-methylamentoflavone	136126990	(Dey et al., 2022)
	Ginkgetin	5271805	
	Sequoiaflavone	5484010	
27	Isovitexin	162350	(Mounika et al., 2010)
	Apigenin	5280443	
28	β-sitosterol	348285530	(Upreti et al., 2021)
	luteoxanthin	12112747	
	violaxanthin	448438	
29	Emetine	10219	(Snoussi et al., 2021)
30	Digitoxigenin	4369270	(Aanouz et al., 2021)
	Crocin	5281233	
	β-Eudesmol	91457	
31	Melatonin	896	(Feitosa et al., 2020)
32	thymoquinone	10281	(Mohideen, 2021)
33	hydroxychloroquine	3652	(Jana et al., 2022)
34	Keto-curcumin	129738071	(Shanmugarajan et al., 2020)
35	Isosilybin	21723007	(Mishra et al., 2021a)

36	chloroquine	2719	(Milenković et al., 2020)
	cinanserin	5475158	
37	isoginkgetin	5318569	(Raj et al., 2022)
	afzelin	5316673	
38	Rutin	5280805	(Jani et al., 2021)
	Swertiapuniside	5487497	
39	ursolic acid	64945	(Halder et al., 2022)
	α-Hederin	319363372	
	hyperoside	5281643	
	nimbaflavone	14492795	
	curcumin	969516	
	epigallocatechin	72277	
	piperine	638024	
	echinocystic acid	73309	
40	Isopomiferin	20055152	(Majeed et al., 2021)
	Lycopene	446925	
	Silydianin	11982272	
41	enterodiol	115089	(Rudrapal et al., 2021)
	taxifolin	439533	
	eriodictyol	440735	
	leucopelargonidin	3286789	
	morin	5281670	
	myricetin	5281672	
42	Echinacoside	5281771	(Bharadwaj et al., 2021b)
	Inulin	24763	
43	Coumarylquinic acid	129709901	(Kamaz et al., 2020)
	stigmasterol	5280794	

44	caffeic acid hexoside	6124135	(Bharathi et al., 2022)
	phloretin	4788	
45	myricitrin	5281673	(Abd El-Mordy et al., 2020)
	mearnsitrin	6918652	
	quercetin	5280343	
46	colchicine	6167	(Mostafa et al., 2022)
	piperine	638024	
47	Quercetin	5280343	(Sekiou et al., 2020)
	Hispidulin	5281628	
48	Taraxerol	92097	(Mujwar and Harwansh, 2022)
49	Bonducellpin D	10835061	(Gurung et al., 2020)
	Caesalmin B	12037261	
50	Albireodelphin	6444318	(Rameshkumar et al., 2021)
51	bisdemethoxycurcumin	5315472	(Sharma et al., 2022a)
	demethoxycurcumin	5469424	
	scutellarin	185617	
	quercetin	5280343	
52	desacetylgedunin	3034112	(Baildya et al., 2021)
53	ellagic acid	5281855	(Bahun et al., 2022)
	epigallocatechin gallate	65064	
	resveratrol	445154	
54	amarogentin	115149	(Kar et al., 2022)
55	gallocatechin-3-gallate	5276890	(Ghosh et al., 2021a)
56	Tenuifolin	21588226	(Prasanth et al.,

	Pavetannin C1	16165472	2021b)
57	Amentoflavone	5281600	(Chandra et al., 2022)
	Baicalin	64982	
58	Clemastanin B	10009802	(Sureja et al., 2022)
	savinin	5281867	
59	α-gurjunene	521243	(Muhammad et al., 2020)
	aromadendrene	91354	
60	laurotetanine	267400	(Kumar et al., 2021b)
	ginkgetin	5271805	
61	Cosmosiine	5280704	(Mahmud et al., 2022)
	Pelargonidin-3-O-glucoside	443648	
	Cleomiscosin A	442510	
62	Astragalin	5282102	(Adejoro et al., 2020)
	Nimbaflavone	14492795	
	Kaempferol	5280863	
63	Acetoside	5281800	(Vincent et al., 2020)
	Luteolin 7 -rutinoside	44258082	
	Chebulagic acid	250397	
	Rutin	5280805	
64	Hesperidin	10621	(Tallei et al., 2020)
	Nabiximols	44148067	
	pectolinarin	168849	
	Rhoifoiln	5282150	
	epigallocatechin gallate	65064	
65	Fusidic acid	3000226	(Kwofie et al., 2021)
66	Ellagic Acid	5281855	(Falade et al., 2021)
	Arjunic Acid	15385516	

	Theasapogenol B	185465	
	Euscaphic Acid	471426	
67	Glabridin	124052	(Ngwe Tun et al., 2022)
68	Canthaxanthin	5281227	(Karpiński et al., 2021)
	Astaxanthin	5281224	
	Flavoxanthin	5281238	
69	Shatavarin IX	101847690	(Patel et al., 2021)
	Racemoside A	102253062	
	Withanoside V	10700345	
70	Salvianolic acid A	5281793	(Ibrahim et al., 2020b)
	curcumin	969516	
71	Amentoflavone	5281600	(Lokhande et al., 2022)
	Agathisflavone	5281599	
72	Taxifolin	439533	(Al-Karmalawy et al., 2021)
	Pectolinarigenin	5320438	
	Tangeretin	68077	
73	Taxifolin	439533	(Gogoi et al., 2021)
	Eriodictyol	440735	
74	Withanolide R	101281364	(Parida et al., 2020)
75	Legalon	5213	(Mishra et al., 2021b)
	Isosilybin	21723007	
76	Neoastilbin	442437	(Naik et al., 2020)
	Astilbin	119258	
77	Azadirachtin H	16722121	(Shadrack et al., 2021)
	Margocin	21632833	
78	Mulberroside A	6443484	(Sharma et al.,

			2022b)
79	Rosmanol	13966122	(Umesh et al., 2021)
80	boceprevir	10324367	(Jan et al., 2021)
81	Catechin	9064	(Yusuf et al., 2022)
	Quercetin	5280343	
82	Dorsilurin E	15478906	(Jiménez-Avalos et al., 2021)
83	Arabic Acid	122045	(Dwarka et al., 2020)
	L-canavanine	439202	
	Uzarin	20055063	
84	Fortunellin	5317385	(Agrawal et al., 2022)
85	Pentagalloylglucose	65238	(Suručić et al., 2022)
86	Plitidepsin	9812534	(El Hassab et al., 2022)
87	Theaflavin digallate	3589471	(Fertier et al., 2020)
88	hesperidin	10621	(Basu et al., 2020)
	emodin	3220	
	chrysin	5281607	
89	ZINC70698944	95372568	(Motwalli and Alazmi, 2021)
	ZINC2121036	1776037	
90	Limolin	179651	(Vardhan and Sahoo, 2022)
	Gedunin	12004512	
	Eribulin	11354606	
91	Podocarpusflavon-B	5320646	(Bharadwaj et al., 2021a)
92	gedunin	12004512	(Garg et al.,

	epoxyazadiradione	49863985	2020a)
	nimbin	108058	
	ginsenosides	3086007	
93	Carnosol	442009	(Das et al., 2023)
	Arjunglucoside-I	14658050	
	Rosmanol	13966122	
94	theaflavin (TF1)	620854	(Singh et al., 2021b)
	Hesperidin	10621	
	myricetin	5281672	
	quercetagetin	5281680	
95	Nictoflorin	5318767	(Srivastava et al., 2022)
	Astragalin	5282102	
	Lupeol	259846	
	Aloenin	162305	
	Aloesin	160190	
	Berberine	2353	
	Sitosterol	222284	
	Ursolic acid	64945	
96	Apigenin	5280443	(Farhat et al., 2022)
97	taraxerol	92097	(Kar et al., 2021)
98	Ellagitannins	53790482	(Mauriz and Lechuga, 2021)
99	octacosanol,	68406	(Chowdhury, 2020)
100	Withanoside V	10700345	(Tripathi et al., 2020b)
101	oxopowelline	626982	(Saliu et al., 2021)
102	Quinine	3034034	(Gandhi et al.,

			2022)
103	warfarin	54678486	(Özdemir et al., 2022)
104	Baicalein	5281605	(Wang et al., 2021)
105	arbidol	131411	(Muhammad et al., 2021)
106	cepharanthine	10206	(Pal et al., 2022)
107	Acetyleugenol	7136	(Sharma and Sharma, 2022)
108	Corilagin	73568	(Yang et al., 2021)
109	Geraniaceae	634578	(Arokiyaraj et al., 2020)
110	Hesperidin	10621	(Owis et al., 2021)
111	silvestrol	11787114	(Liu et al., 2022)
112	friedelin	11787114	(Anywar et al., 2021)
113	4-hydroxyisolonchocarpin	5321800	(Ghosh et al., 2021b)
114	acaciin	5317025	(Mohamad et al., 2021)
115	theaflavin	135403798	(Singh et al., 2022)
116	gamma-terpinene	7461	(Perera et al., 2022)
117	Alpinetin	154279	(Bahadur Gurung et al., 2022)
118	Epicatechin	72276	(Al-Shuhaib et al., 2022)
119	Amentoflavone	5281600	(Zhao et al., 2022)
120	Nimbolin A	6443004	(Borkotoky and

			Banerjee, 2021)
121	sesamolin	101746	(Allam et al., 2021)
122	Schizanthine	70698234	(Alfaro et al., 2020)
123	Tannic acid	16129778	(Wang et al., 2020)
124	fortunellin	5317385	(Panagiotopoulos et al., 2021)
	rhoifolin	5282150	
	apiin	5280746	
125	Luteolin-7-glucoside-3′-glucuronide	44258136	(Prasanth et al., 2021a)
126	Pentagalloylglucose	65238	(Mustafa et al., 2022)
	Shephagenin	10373162	
	Isoacteoside	6476333	
	Isoquercitrin	5280804	
	Kappa-Carrageenan	11966249	
	Dolabellin	388132	
127	3-galloylcatechin	367141	(Iheagwam and Rotimi, 2020)
	proanthocyanidin B1	11250133	
	luteolin 7-galactoside	5488493	
128	Camptothecin	24360	(Mandal, 2021)
	Resveratrol	445154	
	Quercetin	5280343	
	Genistein	5280961	
129	Barrigenol	11843846	(Sharma et al., 2021)
	Kaempferol	5280863	
	Myricetin	5281672	
130	Andrographolide	5318517	(Enmozhi et al.,

			2020)
131	curcumin	969516	(Kushari et al., 2022)
	apigenin	5280443	
	chrysophanol	10208	
	gingerol	442793	
132	Bergenin	66065	(Sharbidre et al., 2021)
	beta-Sitosterol	222284	
	Centaurein	5489090	
	Luteolin	5280445	
133	eugenin	10189	(Saraswat et al., 2021)
	amentoflavone	5281600	
	silymarin	5213	
	amoxycillin	33613	
	curcumin	969516	
134	momordicine	14807332	(Ogidigo et al., 2022)
	margolonone	189726	
	nimbandiol	157277	
	17-hydroxyazadiradione	52951892	
135	Isorhoifolin	9851181	(Yañez et al., 2021b)
136	tribuloside	10175330	(Mishra et al., 2021c)
	isosilybin	21723007	
137	Rhamnocitrin	5320946	(Johnson et al., 2021)
	Isokaempferide	5280862	
	Kaempferol	5280863	
138	cryptomisrine	10600127	(Borquaye et al., 2020)
	cryptospirolepine	9914081	
	cryptoquindoline	103636437	

	biscryptolepine	10457065	
139	α-amyrin	73170	(Ali et al., 2020)
140	Withanolides	11049407	(Puttaswamy et al., 2020)
	Pseudojervine	16398499	
	Kamalachalcone	101721039	
141	theaflavin digallate	3589471	(Peele et al., 2020)
142	Neoruscogenin	9910474	(Isa et al., 2022)
143	*cis*-Annonacin	10698767	(Prasad et al., 2021)
144	pyrazolopyrimidinone	53630237	(Horchani et al., 2022)
145	isoiguesterin	11373102	(Gyebi et al., 2021)
146	Siphonaxanthin	5380124	(Yim et al., 2021)
147	Vidarabine	21704	(Eissa et al., 2021)
148	Demeclocycline	54680690	(Poochi et al., 2020)
	Lycopene	446925	
149	Gartanin	5281633	(Mahmud et al., 2021e)
	Robinetin	5281692	
150	Piperine	638024	(Rout et al., 2022)
151	Apigenin-7-O-glucoside	5280704	(Benhander and Abdusalam, 2022)
	Kaempferol-7-O-rutinoside	102225228	
	Kaempferol-3-O-glucuronoside	14185731	
152	pelargonidin-3-glucoside	3080714	(Messaoudi et al., 2021)
153	Nicotiflorin	5318767	(da Silva et al., 2020)
	Calceolarioside B	5273567	(Adem et al.,

154	vitexfolin A	10458788	2021b)
	Scrophuloside B	11712581	
	Nelfinavir	64143	
155	piperidine-4-carboxamide	3772	(Jairajpuri et al., 2021)
156	Tomatidine	65576	(Zrieq et al., 2021)
	Patchouli Alcohol	10955174	
157	Carinol	586373	(Mahmud et al., 2021f)
	Albanin	5830135	
	Myricetin	5281672	
158	Indole	798	(Kumar et al., 2021a)
	Arbidol	131411	
	Delavirdine	441386	
159	Curcumin	969516	(Singh et al., 2021a)
	diacetylcurcumin	6441419	
160	taraxerol	92097	(Mondal et al., 2022)
	withanolide A	11294368	
	Withametelin	364746	
	daturaolone	122859	
161	luteolin	5280445	(Shawan et al., 2021)
	abyssinone II	10064832	
162	qingdainone	3035728	(Vivek-Ananth, R.P. .; Abhijit, R.; Nithin and To, Inhibitors of Human Proteases Key to SARS-CoV-2 infection. Molecules. 2020, 25, 2020)
	edgeworoside	101835682	
	adlumidine	120734	
	ararobinol	438692	
	(+)-oxoturkiyenine	189308	

163	α-hederin	73296	(Mir et al., 2022)
	dithymoquinone	398941	
	nigellicine	11402337	
	nigellidine	136828302	
164	6-demethoxy-4`-O-capillarsine		(Suleimen et al., 2022)
	tenuflorin C		
165	Brazilein	6453902	(Linda Laksmiani et al., 2020)
	brazilin	73384	
166	Saponarin	441381	(Mulpuru and Mishra, 2021)
167	neohesperidin	442439	(Al-Sanea et al., 2021)
168	artemisinin	68827	(Rolta et al., 2021)
169	Withanoside X	101168807	(Chikhale et al., 2021)
170	Columbin	442015	(Thakkar et al., 2021)
	Tinosporide	442068	
	N-trans-feruloyl-tyramine-diacetate		
	Amritoside C, Amritoside B, Amritoside A	73981613	
	Tinocordifolin	100926540	
	Palmatoside G	184515	
	Palmatoside F	180932	
	Maslinic acids	73659	
171	luteolin	5280445	(El-Mageed et al., 2021)
	mundulinol	10363971	
172	hesperidin	10621	(Khater et al., 2021)

173	jezonofol	46226510	(Elsbaey et al., 2021)
	scirpusin A	5458896	
	cassigarol G	10005549	
174	Hesperidin	10621	(Adem et al., 2022)
	rutin	5280805	
	diosmin	5281613	
175	naringin	442428	(Jain et al., 2021a)
176	Glycyrrhizoflavone	5317764	(Elkaeed et al., 2022)
	Arctigenin	64981	
	Thiangazole	6451119	
177	betacyanin	6324775	(Tallei et al., 2021)
178	epicatechin	72276	(Gupta et al., 2022)
	hesperidin	10621	
	mangiferin	5281647	
179	Cepharanthine	10206	(Jain et al., 2021b)
180	withacoagulin H	71524298	(Verma et al., 2021)
	withanolide A	11294368	
181	isovitexin	162350	(Zothantluanga et al., 2021)
182	Hydroxychloroquine	3652	(Abdelrheem et al., 2020)
	Flavonoid	122792	
	Loliolide	100332	
	Hexadecanoic acid	985	
	Caulerpin	5326018	
183	Dieckol	3008868	(Protein and Strain, 2021)
	Nigricanoside A	102102791	
	Nigricanoside A dimethyl ester	101503499	
184	Nelfinavir	64143	(Mpiana et al., 2020)
185	Nelfinavir	64143	(Khaerunnisa et

	Pitavastatin	5282452	al., 2020)
	Perampanel	9924495	
	Praziquantel	4891	
186	Chalcone	637760	(17.Acta Crystallographica Section C - 2020 - Alsafi - First COVID-19 molecular docking with a chalcone-based compound.pdf)
187	Favipiravir	492405	(Emon et al., 2021)
	Theaflavin	135403798	
	Kaempferol	5280863	
	Limonene	22311	
	Sabinene	18818	
	Piperine	638024	
	Catechin	9064	
188	Methylophiopogonone A	10065830	(Harisna et al., 2021)
	3′-Methoxydaidzin	10527347	
	Genistin	5281377	
189	Methoxylated phenol	7519	(El-Hawary et al., 2022)
190	hopeaphenol	495605	(Tietjen et al., 2021)
	Vatalbinoside A<br>Vaticanol B<br>Resveratrol	46938651<br>10010985<br>445154	
191	hydroxychloroquine	3652	(Vijayakumar et al., 2022)
	Andrographolide<br>Neoandrographolide<br>14-Deoxyandrographolide	5318517<br>9848024<br>11624161	
192	Flavonoids	103905853	(Saakre et al., 2021)
	quercetin	5280343	
193	Folic acid	135398658	(Benarous et al.,

	Hispidin<br>Curcumin	54685921<br>969516	2022)
194	Nicotianamine	9882882	(Illian et al., 2021)
195	Hydrocinnamic acid	107	(Mahmud et al., 2021d)
	Phenethyl alcohol	6054	
	Dihydroartemisinin	3000518	
196	Flemichin A	42608042	(Mahmud et al., 2021c)
	Delta-Oleanolic acid	101768915	
	Emodin 1-O-beta-D-glucoside	5319333	
197	Medicagol	5319322	(Mahmud et al., 2021b)
	Faradiol	9846222	
	Flavanthrin	102004681	
198	28-demethyl-beta-amyrone	101616676	(Kumar Paul et al., 2022)
	24-Noroleana-3,12-diene	15427754	
	Stigmasterol	5280794	
199	Nimbin	108058	(Garg et al., 2020b)
	Gedunin	12004512	
	Epoxyazadiradione	49863985	
200	Marmin	6450230	(Qazi et al., 2021)
	Malic acid	525	
	Benzylamine	7504	

References

1. Acta Crystallographica Section C - 2020 - Alsafi - First COVID-19 molecular docking with a chalcone-based compound.pdf.
2. Aanouz, I., Belhassan, A., El-Khatabi, K., Lakhlifi, T., El-ldrissi, M., and Bouachrine, M. (2021). Moroccan Medicinal plants as inhibitors against SARS-CoV-2 main protease: Computational investigations. *J. Biomol. Struct. Dyn.* 39, 2971–2979. doi:10.1080/07391102.2020.1758790.
3. Abd El-Mordy, F. M., El-Hamouly, M. M., Ibrahim, M. T., El-Rheem, G. A., Aly, O. M., Abd El-Kader, A. M., et al. (2020). Inhibition of SARS-CoV-2 main protease by phenolic compounds fromManilkara hexandra(Roxb.) Dubard assisted by metabolite profiling andin silicovirtual screening. *RSC Adv.* 10, 32148–32155. doi:10.1039/d0ra05679k.
4. Abdelrheem, D. A., Ahmed, S. A., Abd El-Mageed, H. R., Mohamed, H. S., Rahman, A. A., Elsayed, K. N. M., et al. (2020). The inhibitory effect of some natural bioactive compounds against SARS-CoV-2 main protease: insights from molecular docking analysis and molecular dynamic simulation. *J. Environ. Sci. Heal. - Part A Toxic/Hazardous Subst. Environ. Eng.* 55, 1373–1386. doi:10.1080/10934529.2020.1826192.
5. Adejoro, I. A., Babatunde, D. D., and Tolufashe, G. F. (2020). Molecular docking and dynamic simulations of some medicinal plants compounds against SARS-CoV-2: an in silico study . *J. Taibah Univ. Sci.* 14, 1563–1570. doi:10.1080/16583655.2020.1848049.
6. Adem, Ş., Eyupoglu, V., Ibrahim, I. M., Sarfraz, I., Rasul, A., Ali, M., et al. (2022). Multidimensional in silico strategy for identification of natural polyphenols-based SARS-CoV-2 main protease (Mpro) inhibitors to unveil a hope against COVID-19. *Comput. Biol. Med.* 145. doi:10.1016/j.compbiomed.2022.105452.

7. Adem, Ş., Eyupoglu, V., Sarfraz, I., Rasul, A., Zahoor, A. F., Ali, M., et al. (2021a). Caffeic acid derivatives (CAFDs) as inhibitors of SARS-CoV-2: CAFDs-based functional foods as a potential alternative approach to combat COVID-19. *Phytomedicine* 85. doi:10.1016/j.phymed.2020.153310.
8. Adem, Ş., Eyupoglu, V., Sarfraz, I., Rasul, A., Zahoor, A. F., Ali, M., et al. (2021b). Caffeic acid derivatives (CAFDs) as inhibitors of SARS-CoV-2: CAFDs-based functional foods as a potential alternative approach to combat COVID-19. *Phytomedicine* 85. doi:10.1016/j.phymed.2020.153310.
9. Agrawal, S., Pathak, E., Mishra, V., Parveen, A., and Mishra, S. (2022). Computational exploration of the dual role of the phytochemical fortunellin: antiviral activities against SARS-CoV-2 and immunomodulatory abilities against the host. *Springer* 1352, 1–13. doi:10.1016/j.compbiomed.2022.106049.
10. Al-Bustany, H. A., Ercan, S., Ince, E., and Pirinccioglu, N. (2022). Investigation of angucycline compounds as potential drug candidates against SARS Cov-2 main protease using docking and molecular dynamic approaches. *Mol. Divers.* 26, 293–308. doi:10.1007/s11030-021-10219-1.
11. Al-Karmalawy, A. A., Farid, M. M., Mostafa, A., Ragheb, A. Y., Mahmoud, S. H., Shehata, M., et al. (2021). Naturally available flavonoid aglycones as potential antiviral drug candidates against SARS-CoV-2. *Molecules* 26, 1–11. doi:10.3390/molecules26216559.
12. Al-Sanea, M. M., Abelyan, N., Abdelgawad, M. A., Musa, A., Ghoneim, M. M., Al-Warhi, T., et al. (2021). Strawberry and ginger silver nanoparticles as potential inhibitors for sars-cov-2 assisted by in silico modeling and metabolic profiling. *Antibiotics* 10. doi:10.3390/antibiotics10070824.
13. Al-Shuhaib, M. B. S., Hashim, H. O., and Al-Shuhaib, J. M. B. (2022). Epicatechin is a promising novel inhibitor of SARS-CoV-2 entry by disrupting interactions between angiotensin-converting enzyme type 2 and the viral receptor binding domain: A computational/simulation study. *Comput. Biol. Med.* 141, 105155. doi:10.1016/j.compbiomed.2021.105155.
14. Alfaro, M., Alfaro, I., and Angel, C. (2020). Identification of potential inhibitors of SARS-CoV-2 papain-like protease from tropane alkaloids from Schizanthus porrigens: A molecular docking study. *Chem. Phys. Lett.* 761, 138068. doi:10.1016/j.cplett.2020.138068.
15. Ali, M. C., Nur, A. J., Khatun, M. S., Dash, R., Rahman, M. M., and Karim, M. M. (2020). Identification of potential sars-cov-2 main protease inhibitors from ficus carica latex: An in-silico approach. *J. Adv. Biotechnol. Exp. Ther.* 3, 57–67. doi:10.5455/JABET.2020.D157.
16. Allam, A. E., Amen, Y., Ashour, A., Assaf, H. K., Hassan, H. A., Abdel-Rahman, I. M., et al. (2021). In silico study of natural compounds from sesame against COVID-19 by targeting Mpro, PLpro and RdRp. *RSC Adv.* 11, 22398–22408. doi:10.1039/d1ra03937g.
17. Anywar, G., Akram, M., and Chishti, M. A. (2021). African and Asian Medicinal Plants as a Repository for Prospective Antiviral Metabolites Against HIV-1 and SARS CoV-2: A Mini Review. *Front. Pharmacol.* 12, 1–10. doi:10.3389/fphar.2021.703837.
18. Arokiyaraj, S., Stalin, A., Kannan, B. S., and Shin, H. (2020). Geranii herba as a potential inhibitor of SARS-CoV-2 main 3CLpro, spike RBD, and regulation of unfolded protein response: An in silico approach. *Antibiotics* 9, 1–16. doi:10.3390/antibiotics9120863.
19. Bahadur Gurung, A., Ajmal Ali, M., Al-Hemaid, F., El-Zaidy, M., and Lee, J. (2022). In silico analyses of major active constituents of fingerroot (Boesenbergia rotunda) unveils inhibitory activities against SARS-CoV-2 main protease enzyme. *Saudi J. Biol. Sci.* 29, 65–74. doi:10.1016/j.sjbs.2021.11.053.
20. Bahun, M., Jukić, M., Oblak, D., Kranjc, L., Bajc, G., Butala, M., et al. (2022). Inhibition of the SARS-CoV-2 3CLpro main protease by plant polyphenols. *Food Chem.* 373. doi:10.1016/j.foodchem.2021.131594.
21. Baildya, N., Khan, A. A., Ghosh, N. N., Dutta, T., and Chattopadhyay, A. P. (2021). Screening of potential drug from Azadirachta Indica (Neem) extracts for SARS-CoV-2: An insight from molecular docking and MD-simulation studies. *J. Mol. Struct.* 1227, 129390. doi:10.1016/j.molstruc.2020.129390.
22. Basu, A., Sarkar, A., and Maulik, U. (2020). Computational approach for the design of potential spike protein binding natural compounds in SARS- CoV2. 1–22.
23. Benarous, L., Benarous, K., Muhammad, G., and Ali, Z. (2022). Deep learning application detecting SARS-CoV-2 key enzymes inhibitors. *Cluster Comput.* 6. doi:10.1007/s10586-022-03656-6.
24. Benhander, G. M., and Abdusalam, A. A. A. (2022). Identification of Potential Inhibitors of SARS-CoV-2 Main Protease from Allium roseum L. Molecular Docking Study. *Chem. Africa* 5, 57–67. doi:10.1007/s42250-021-00296-y.
25. Bharadwaj, S., Dubey, A., Yadava, U., Mishra, S. K., Kang, S. G., and Dwivedi, V. D. (2021a). Exploration of natural compounds with anti-SARS-CoV-2 activity via inhibition of SARS-CoV-2 Mpro. *Brief. Bioinform.* 22, 1361–1377. doi:10.1093/bib/bbaa382.
26. Bharadwaj, S., El-Kafrawy, S. A., Alandijany, T. A., Bajrai, L. H., Shah, A. A., Dubey, A., et al. (2021b). Structure-based identification of natural products as sars-cov-2 mpro antagonist from echinacea angustifolia using computational approaches. *Viruses* 13. doi:10.3390/v13020305.
27. Bharathi, M., Sivamaruthi, B. S., Kesika, P., Thangaleela, S., and Chaiyasut, C. (2022). In Silico Screening of Bioactive Compounds of Representative Seaweeds to Inhibit SARS-CoV-2 ACE2-Bound Omicron B.1.1.529 Spike Protein Trimer. *Mar. Drugs* 20, 1–18. doi:10.3390/md20020148.
28. Bhardwaj, A., Sharma, S., and Singh, S. K. (2022). Molecular Docking Studies to Identify Promising Natural Inhibitors Targeting SARS-CoV-2 Nsp10- Nsp16 Protein Complex. *Turkish J. Pharm. Sci.* 19, 93–100. doi:10.4274/tjps.galenos.2021.56957.
29. Bhardwaj, V. K., Singh, R., Sharma, J., Rajendran, V., Purohit, R., and Kumar, S. (2021). Bioactive Molecules of Tea as Potential Inhibitors for RNA-Dependent RNA Polymerase of SARS-CoV-2. *Front. Med.* 8, 1–11. doi:10.3389/fmed.2021.684020.

30. Borkotoky, S., and Banerjee, M. (2021). A computational prediction of SARS-CoV-2 structural protein inhibitors from Azadirachta indica (Neem). *J. Biomol. Struct. Dyn.* 39, 4111–4121. doi:10.1080/07391102.2020.1774419.
31. Borquaye, L. S., Gasu, E. N., Ampomah, G. B., Kyei, L. K., Amarh, M. A., Mensah, C. N., et al. (2020). Alkaloids from Cryptolepis sanguinolenta as Potential Inhibitors of SARS-CoV-2 Viral Proteins: An in Silico Study. *Biomed Res. Int.* 2020. doi:10.1155/2020/5324560.
32. Chandra, A., Chaudhary, M., Qamar, I., Singh, N., and Nain, V. (2022). In silico identification and validation of natural antiviral compounds as potential inhibitors of SARS-CoV-2 methyltransferase. *J. Biomol. Struct. Dyn.* 40, 6534–6544. doi:10.1080/07391102.2021.1886174.
33. Chikhale, R. V., Gurav, S. S., Patil, R. B., Sinha, S. K., Prasad, S. K., Shakya, A., et al. (2021). Sars-cov-2 host entry and replication inhibitors from Indian ginseng: an in-silico approach. *J. Biomol. Struct. Dyn.* 39, 4510–4521. doi:10.1080/07391102.2020.1778539.
34. Chowdhury, P. (2020). In silico investigation of phytoconstituents from Indian medicinal herb 'Tinospora cordifolia (giloy)' against SARS-CoV-2 (COVID-19) by molecular dynamics approach. *J. Biomol. Struct. Dyn.* doi:10.1080/07391102.2020.1803968.
35. da Silva, F. M. A., da Silva, K. P. A., de Oliveira, L. P. M., Costa, E. V., Koolen, H. H. F., Pinheiro, M. L. B., et al. (2020). Flavonoid glycosides and their putative human metabolites as potential inhibitors of the sars-cov-2 main protease (Mpro) and rna-dependent rna polymerase (rdrp). *Mem. Inst. Oswaldo Cruz* 115, 1–8. doi:10.1590/0074-02760200207.
36. Das, C., Das, D., and Mattaparthi, V. S. K. (2023). Computational Investigation on the Efficiency of Small Molecule Inhibitors Identified from Indian Spices against SARS-CoV-2 Mpro. *Biointerface Res. Appl. Chem.* 13. doi:10.33263/BRIAC133.235.
37. Das, S., Singh, A., Samanta, S. K., and Singha Roy, A. (2022). Naturally occurring anthraquinones as potential inhibitors of SARS-CoV-2 main protease: an integrated computational study. *Biologia (Bratisl).* 77, 1121–1134. doi:10.1007/s11756-021-01004-4.
38. Dey, D., Hossain, R., Biswas, P., Paul, P., Islam, M. A., Ema, T. I., et al. (2022). Amentoflavone derivatives significantly act towards the main protease (3CLPRO/MPRO) of SARS-CoV-2: in silico admet profiling, molecular docking, molecular dynamics simulation, network pharmacology. *Mol. Divers.* doi:10.1007/s11030-022-10459-9.
39. Dwarka, D., Agoni, C., Mellem, J. J., Soliman, M. E., and Baijnath, H. (2020). Identification of potential SARS-CoV-2 inhibitors from South African medicinal plant extracts using molecular modelling approaches. *South African J. Bot.* 133, 273–284. doi:10.1016/j.sajb.2020.07.035.
40. Eissa, I. H., Khalifa, M. M., Elkaeed, E. B., Hafez, E. E., Alsfouk, A. A., and Metwaly, A. M. (2021). In silico exploration of potential natural inhibitors against sars-cov-2 nsp10. *Molecules* 26. doi:10.3390/molecules26206151.
41. El-Hawary, S. S., Ali, T. F. S., Abo El-Ela, S. O., Elwekeel, A., Abdelmohsen, U. R., and Owis, A. I. (2022). Secondary metabolites of Livistona decipiens as potential inhibitors of SARS-CoV-2. *RSC Adv.* 12, 19505–19511. doi:10.1039/d2ra01306a.
42. El-Mageed, H. R. A., Abdelrheem, D. A., Rafi, M. O., Sarker, M. T., Al-Khafaji, K., Hossain, M. J., et al. (2021). In Silico Evaluation of Different Flavonoids from Medicinal Plants for Their Potency against SARS-CoV-2. *Biologics* 1, 416–434. doi:10.3390/biologics1030024.
43. El Hassab, M. A., Hemeda, L. R., Elsayed, Z. M., Al-Rashood, S. T., Abdel-Hamid Amin, M. K., Abdel-Aziz, H. A., et al. (2022). Computational Prediction of the Potential Target of SARS-CoV-2 Inhibitor Plitidepsin via Molecular Docking, Dynamic Simulations and MM-PBSA Calculations. *Chem. Biodivers.* 19. doi:10.1002/cbdv.202100719.
44. Elkaeed, E. B., Metwaly, A. M., Alesawy, M. S., Saleh, A. M., Alsfouk, A. A., and Eissa, I. H. (2022). Discovery of Potential SARS-CoV-2 Papain-like Protease Natural Inhibitors Employing a Multi-Phase In Silico Approach. *Life* 12. doi:10.3390/life12091407.
45. Elsbaey, M., Ibrahim, M. A. A., Bar, F. A., and Elgazar, A. A. (2021). Chemical constituents from coconut waste and their in silico evaluation as potential antiviral agents against SARS-CoV-2. *South African J. Bot.* 141, 278–289. doi:10.1016/j.sajb.2021.05.018.
46. Emon, N. U., Alam, M. M., Akter, I., Akhter, S., Sneha, A. A., Irtiza, M., et al. (2021). Virtual screenings of the bioactive constituents of tea, prickly chaff, catechu, lemon, black pepper, and synthetic compounds with the main protease (Mpro) and human angiotensin-converting enzyme 2 (ACE 2) of SARS-CoV-2. *Futur. J. Pharm. Sci.* 7. doi:10.1186/s43094-021-00275-7.
47. Enmozhi, S. K., Raja, K., Sebastine, I., and Joseph, J. (2020). Andrographolide as a potential inhibitor of SARS-CoV-2 main protease: an in silico approach. *J. Biomol. Struct. Dyn.* 0, 1–7. doi:10.1080/07391102.2020.1760136.
48. Faisal, S., Lal Badshah, S., Kubra, B., Sharaf, M., Emwas, A. H., Jaremko, M., et al. (2022). Computational study of sars-cov-2 rna dependent rna polymerase allosteric site inhibition. *Molecules* 27, 1–15. doi:10.3390/molecules27010223.
49. Falade, V. A., Adelusi, T. I., Adedotun, I. O., Abdul-Hammed, M., Lawal, T. A., and Agboluaje, S. A. (2021). In silico investigation of saponins and tannins as potential inhibitors of SARS-CoV-2 main protease (Mpro). *Silico Pharmacol.* 9, 1–15. doi:10.1007/s40203-020-00071-w.
50. Farhat, A., Ben Hlima, H., Khemakhem, B., Ben Halima, Y., Michaud, P., Abdelkafi, S., et al. (2022). Apigenin analogues as SARS-CoV-2 main protease inhibitors: In-silico screening approach. *Bioengineered* 13, 3350–3361. doi:10.1080/21655979.2022.2027181.
51. Feitosa, E. L., Júnior, F. T. D. S. S., Neto, J. A. D. O. N., Matos, L. F. L., Moura, M. H. D. S., Rosales, T. O., et al. (2020). Covid-19: Rational discovery of the therapeutic potential of melatonin as a sars-cov-2 main protease inhibitor. *Int. J. Med. Sci.* 17, 2133–2146. doi:10.7150/ijms.48053.

52. Ferdous, N., Reza, M. N., Islam, M. S., Hossain Emon, M. T., Mohiuddin, A. K. M., and Hossain, M. U. (2021). Newly designed analogues from SARS-CoV inhibitors mimicking the druggable properties against SARS-CoV-2 and its novel variants. *RSC Adv.* 11, 31460–31476. doi:10.1039/d1ra04107j.
53. Fertier, A., Montarnal, A., Truptil, S., and Bénaben, F. (2020). Jo ur na l P re Jo ur l P re. *Decis. Support Syst.*, 113260. doi:10.1016/j.compbiomed.2022.106125.
54. Gandhi, A. J., Rupareliya, J. D., Shukla, V. J., Donga, S. B., and Acharya, R. (2022). An ayurvedic perspective along with in silico study of the drugs for the management of SARS-CoV-2. *J. Ayurveda Integr. Med.* 13, 100343. doi:10.1016/j.jaim.2020.07.002.
55. Garg, S., Anand, A., Lamba, Y., and Roy, A. (2020a). Molecular docking analysis of selected phytochemicals against SARS-CoV-2 Mpro receptor. *Vegetos* 33, 766–781. doi:10.1007/s42535-020-00162-1.
56. Garg, S., Anand, A., Lamba, Y., and Roy, A. (2020b). Molecular docking analysis of selected phytochemicals against SARS-CoV-2 Mpro receptor. *Vegetos* 33, 766–781. doi:10.1007/s42535-020-00162-1.
57. Garg, S., and Roy, A. (2020). In silico analysis of selected alkaloids against main protease (Mpro) of SARS-CoV-2. *Chem. Biol. Interact.* 332, 109309. doi:10.1016/j.cbi.2020.109309.
58. Ghosh, R., Chakraborty, A., Biswas, A., and Chowdhuri, S. (2021a). Evaluation of green tea polyphenols as novel corona virus (SARS CoV-2) main protease (Mpro) inhibitors–an in silico docking and molecular dynamics simulation study. *J. Biomol. Struct. Dyn.* 39, 4362–4374. doi:10.1080/07391102.2020.1779818.
59. Ghosh, R., Chakraborty, A., Biswas, A., and Chowdhuri, S. (2021b). Identification of polyphenols from Broussonetia papyrifera as SARS CoV-2 main protease inhibitors using in silico docking and molecular dynamics simulation approaches. *J. Biomol. Struct. Dyn.* 39, 6747–6760. doi:10.1080/07391102.2020.1802347.
60. Gogoi, N., Chowdhury, P., Goswami, A. K., Das, A., Chetia, D., and Gogoi, B. (2021). Computational guided identification of a citrus flavonoid as potential inhibitor of SARS-CoV-2 main protease. *Mol. Divers.* 25, 1745–1759. doi:10.1007/s11030-020-10150-x.
61. Gupta, S., Singh, V., Varadwaj, P. K., Chakravartty, N., Katta, A. V. S. K. M., Lekkala, S. P., et al. (2022). Secondary metabolites from spice and herbs as potential multitarget inhibitors of SARS-CoV-2 proteins. *J. Biomol. Struct. Dyn.* 40, 2264–2283. doi:10.1080/07391102.2020.1837679.
62. Gurung, A. B., Ali, M. A., Lee, J., Farah, M. A., and Al-Anazi, K. M. (2020). Unravelling lead antiviral phytochemicals for the inhibition of SARS-CoV-2 Mpro enzyme through in silico approach. *Life Sci.* 255, 117831. doi:https://doi.org/10.1016/j.lfs.2020.117831.
63. Gyebi, G. A., Ogunyemi, O. M., Ibrahim, I. M., Ogunro, O. B., Adegunloye, A. P., and Afolabi, S. O. (2021). SARS-CoV-2 host cell entry: an in silico investigation of potential inhibitory roles of terpenoids. *J. Genet. Eng. Biotechnol.* 19. doi:10.1186/s43141-021-00209-z.
64. Haddad, M., Gaudreault, R., Sasseville, G., Nguyen, P. T., Wiebe, H., Van De Ven, T., et al. (2022). Molecular Interactions of Tannic Acid with Proteins Associated with SARS-CoV-2 Infectivity. *Int. J. Mol. Sci.* 23. doi:10.3390/ijms23052643.
65. Halder, P., Pal, U., Paladhi, P., Dutta, S., Paul, P., Pal, S., et al. (2022). Evaluation of potency of the selected bioactive molecules from Indian medicinal plants with MPro of SARS-CoV-2 through in silico analysis. *J. Ayurveda Integr. Med.* 13, 100449. doi:10.1016/j.jaim.2021.05.003.
66. Harisna, A. H., Nurdiansyah, R., Syaifie, P. H., Nugroho, D. W., Saputro, K. E., Firdayani, et al. (2021). In silico investigation of potential inhibitors to main protease and spike protein of SARS-CoV-2 in propolis. *Biochem. Biophys. Reports* 26, 100969. doi:10.1016/j.bbrep.2021.100969.
67. Hejazi, I. I., Beg, M. A., Imam, M. A., Athar, F., and Islam, A. (2021). Glossary of phytoconstituents: Can these be repurposed against SARS CoV-2? A quick in silico screening of various phytoconstituents from plant Glycyrrhiza glabra with SARS CoV-2 main protease. *Food Chem. Toxicol.* 150, 112057. doi:10.1016/j.fct.2021.112057.
68. Horchani, M., Heise, N. V., Csuk, R., Ben Jannet, H., Harrath, A. H., and Romdhane, A. (2022). Synthesis and In Silico Docking Study towards M-Pro of Novel Heterocyclic Compounds Derived from Pyrazolopyrimidinone as Putative SARS-CoV-2 Inhibitors. *Molecules* 27. doi:10.3390/molecules27165303.
69. Hossain, R., Islam, M. T., Ray, P., Jain, D., Saikat, A. S. M., Nahar, L., et al. (2021). Amentoflavone, New Hope against SARS-CoV-2: An Outlook through its Scientific Records and an in silico Study. *Pharmacognosy Res.* 13, 149–157. doi:10.5530/pres.13.3.7.
70. Ibrahim, M. A. A., Abdelrahman, A. H. M., Hussien, T. A., Badr, E. A. A., Mohamed, T. A., El-Seedi, H. R., et al. (2020a). In silico drug discovery of major metabolites from spices as SARS-CoV-2 main protease inhibitors. *Comput. Biol. Med.* 126, 104046. doi:10.1016/j.compbiomed.2020.104046.
71. Ibrahim, M. A. A., Abdelrahman, A. H. M., Hussien, T. A., Badr, E. A. A., Mohamed, T. A., El-Seedi, H. R., et al. (2020b). In silico drug discovery of major metabolites from spices as SARS-CoV-2 main protease inhibitors. *Comput. Biol. Med.* 126, 104046. doi:10.1016/j.compbiomed.2020.104046.
72. Iheagwam, F. N., and Rotimi, S. O. (2020). Computer-Aided Analysis of Multiple SARS-CoV-2 Therapeutic Targets: Identification of Potent Molecules from African Medicinal Plants. *Scientifica (Cairo).* 2020. doi:10.1155/2020/1878410.
73. Illian, D. N., Siregar, E. S., Sumaiyah, S., Utomo, A. R., Nuryawan, A., and Basyuni, M. (2021). Potential compounds from several Indonesian plants to prevent SARS-CoV-2 infection: A mini-review of SARS-CoV-2 therapeutic targets. *Heliyon* 7, e06001. doi:10.1016/j.heliyon.2021.e06001.
74. In, S.-A., Study, S., Singh, R., Gautam, A., Chandel, S., Ghosh, A., et al. Protease Inhibitory E ff ect of Natural Polyphenolic. 1–18.
75. Isa, M. A., Mustapha, A., Qazi, S., Raza, K., Allamin, I. A., Ibrahim, M. M., et al. (2022). In silico molecular docking and molecular dynamic simulation of potential inhibitors of 3C-like main proteinase (3CLpro) from severe acute respiratory

syndrome coronavirus-2 (SARS-CoV-2) using selected african medicinal plants. *Adv. Tradit. Med.* 22, 107–123. doi:10.1007/s13596-020-00523-w.

76. Jain, A. S., Sushma, P., Dharmashekar, C., Beelagi, M. S., Prasad, S. K., Shivamallu, C., et al. (2021a). In silico evaluation of flavonoids as effective antiviral agents on the spike glycoprotein of SARS-CoV-2. *Saudi J. Biol. Sci.* 28, 1040–1051. doi:10.1016/j.sjbs.2020.11.049.
77. Jain, D., Hossain, R., Khan, R. A., Dey, D., Sheikh, B., and Rahman, M. (2021b). Computer-aided Evaluation of Anti-SARS-CoV-2 (3-chymotrypsin-like Protease and Transmembrane Protease Serine 2 Inhibitors) Activity of Cepharanthine: An In silico Approach. *Biointerface Res. Appl. Chem.* 12, 768–780. doi:10.33263/briac121.768780.
78. Jairajpuri, D. S., Hussain, A., Nasreen, K., Mohammad, T., Anjum, F., Tabish Rehman, M., et al. (2021). Identification of natural compounds as potent inhibitors of SARS-CoV-2 main protease using combined docking and molecular dynamics simulations. *Saudi J. Biol. Sci.* 28, 2423–2431. doi:10.1016/j.sjbs.2021.01.040.
79. Jan, J. T., Cheng, T. J. R., Juang, Y. P., Ma, H. H., Wu, Y. T., Yang, W. Bin, et al. (2021). Identification of existing pharmaceuticals and herbal medicines as inhibitors of SARS-CoV-2 infection. *Proc. Natl. Acad. Sci. U. S. A.* 118, 1–8. doi:10.1073/pnas.2021579118.
80. Jana, A., Roy, T., Layek, S., Ghosal, S., and Banerjee, D. R. (2022). Computational Investigation on Natural Quinazoline Alkaloids as Potential Inhibitors of the Main Protease (M pro) of SARS-CoV-2. *J. Comput. Biophys. Chem.* 21, 65–82. doi:10.1142/S2737416522500053.
81. Jani, V., Koulgi, S., Uppuladinne, V. N. M., Sonavane, U., and Joshi, R. (2021). An insight into the inhibitory mechanism of phytochemicals and FDA-approved drugs on the ACE2–Spike complex of SARS-CoV-2 using computational methods. *Chem. Pap.* 75, 4625–4648. doi:10.1007/s11696-021-01680-1.
82. Jiménez-Avalos, G., Vargas-Ruiz, A. P., Delgado-Pease, N. E., Olivos-Ramirez, G. E., Sheen, P., Fernández-Díaz, M., et al. (2021). Comprehensive virtual screening of 4.8 k flavonoids reveals novel insights into allosteric inhibition of SARS-CoV-2 MPRO. *Sci. Rep.* 11, 1–19. doi:10.1038/s41598-021-94951-6.
83. Johnson, T., Adegboyega, A., Ojo, O. A., Jega, A., Iwaloye, O., Ugwah-Oguejiofor, C., et al. (2021). Identification of possible inhibitors of SARS-CoV-2 main protease from some bioactive compounds of artemisia annua: An in Silico Approach. 26 June 2021, PREPRINT (Version 1) available at Research Square [+https://doi.org/10.21203/rs.3.rs-612899/v1+]. *Preprint*, 1–16.
84. Kamaz, Z., Jassani, M. J. A.-, and Haruna, U. (2020). Screening of Common Herbal Medicines as Promising Direct Inhibitors of Sars-Cov-2 in Silico. *Annu. Res. Rev. Biol.* 35, 53–67. doi:10.9734/arrb/2020/v35i830260.
85. Kar, P., Kumar, V., Vellingiri, B., Sen, A., Jaishee, N., Anandraj, A., et al. (2022). Anisotine and amarogentin as promising inhibitory candidates against SARS-CoV-2 proteins: a computational investigation. *J. Biomol. Struct. Dyn.* 40, 4532–4542. doi:10.1080/07391102.2020.1860133.
86. Kar, P., Sharma, N. R., Singh, B., Sen, A., and Roy, A. (2021). Natural compounds from Clerodendrum spp. as possible therapeutic candidates against SARS-CoV-2: An in silico investigation. *J. Biomol. Struct. Dyn.* 39, 4774–4785. doi:10.1080/07391102.2020.1780947.
87. Karpiński, T. M., Kwaśniewski, M., Ozarowski, M., and Alam, R. (2021). In silico studies of selected xanthophylls as potential candidates against SARS-CoV-2 targeting main protease (Mpro) and papain-like protease (PLpro). *Herba Pol.* 67, 1–8. doi:10.2478/hepo-2021-0009.
88. Khaerunnisa, S., Kurniawan, H., Awaluddin, R., Suhartati, S., and Soetjipto, S. (2020). Potential Inhibitor of COVID-19 Main Protease (Mpro) From Several Medicinal Plant Compounds by Molecular Docking Study Molecular Docking, ADME-Toxicity Prediction, and Evaluation of Curcumin Derivative Compound as Inhibitor Inflamation on Rheumathoid Arth. *Preprints*, 1–14. doi:10.20944/preprints202003.0226.v1.
89. Khan, A., Heng, W., Wang, Y., Qiu, J., Wei, X., Peng, S., et al. (2021). In silico and in vitro evaluation of kaempferol as a potential inhibitor of the SARS-CoV-2 main protease (3CLpro). *Phyther. Res.* 35, 2841–2845. doi:10.1002/ptr.6998.
90. Khater, S., Kumar, P., Dasgupta, N., Das, G., Ray, S., and Prakash, A. (2021). Combining SARS-CoV-2 Proofreading Exonuclease and RNA-Dependent RNA Polymerase Inhibitors as a Strategy to Combat COVID-19: A High-Throughput in silico Screening. *Front. Microbiol.* 12, 1–10. doi:10.3389/fmicb.2021.647693.
91. Kumar, A., Loharch, S., Kumar, S., Ringe, R. P., and Parkesh, R. (2021a). Exploiting cheminformatic and machine learning to navigate the available chemical space of potential small molecule inhibitors of SARS-CoV-2. *Comput. Struct. Biotechnol. J.* 19, 424–438. doi:10.1016/j.csbj.2020.12.028.
92. Kumar, N., Singh, A., Gulati, H. K., Bhagat, K., Kaur, K., Kaur, J., et al. (2021b). Phytoconstituents from ten natural herbs as potent inhibitors of main protease enzyme of SARS-COV-2: In silico study. *Phytomedicine Plus* 1, 100083. doi:10.1016/j.phyplu.2021.100083.
93. Kumar Paul, G., Mahmud, S., Aldahish, A. A., Afroze, M., Biswas, S., Briti Ray Gupta, S., et al. (2022). Computational screening and biochemical analysis of Pistacia integerrima and Pandanus odorifer plants to find effective inhibitors against Receptor-Binding domain (RBD) of the spike protein of SARS-Cov-2. *Arab. J. Chem.* 15, 103600. doi:10.1016/j.arabjc.2021.103600.
94. Kushari, S., Hazarika, I., Laloo, D., Kumar, S., and Kalita, J. M. (2022). *An integrated computational approach towards the screening of active plant metabolites as potential inhibitors of SARS - CoV - 2 : an overview*. Springer US doi:10.1007/s11224-022-02066-z.
95. Kwofie, S. K., Broni, E., Asiedu, S. O., Kwarko, G. B., Dankwa, B., Enninful, K. S., et al. (2021). Cheminformatics-based identification of potential novel anti-sars-cov-2 natural compounds of african origin. *Molecules* 26. doi:10.3390/molecules26020406.

96. Linda Laksmiani, N. P., Febryana Larasanty, L. P., Jaya Santika, A. A. G., Andika Prayoga, P. A., Kharisma Dewi, A. A. I., and Kristiara Dewi, N. P. A. (2020). Active compounds activity from the medicinal plants against SARS-CoV-2 using in silico assay. *Biomed. Pharmacol. J.* 13, 873–881. doi:10.13005/BPJ/1953.
97. Liu, Q., Kwan, K. Y., Cao, T., Yan, B., Ganesan, K., Jia, L., et al. (2022). Broad-spectrum antiviral activity of Spatholobus suberectus Dunn against SARS-CoV-2, SARS-CoV-1, H5N1, and other enveloped viruses. *Phyther. Res.* 36, 3232–3247. doi:10.1002/ptr.7452.
98. Lokhande, K., Nawani, N., K. Venkateswara, S., and Pawar, S. (2022). Biflavonoids from Rhus succedanea as probable natural inhibitors against SARS-CoV-2: a molecular docking and molecular dynamics approach. *J. Biomol. Struct. Dyn.* 40, 4376–4388. doi:10.1080/07391102.2020.1858165.
99. Lu, J., Lu, W., Jiang, H., Yang, C., and Dong, X. (2022). Molecular Docking and Dynamics of Phytochemicals From Chinese Herbs With SARS-CoV-2 RdRp. *Nat. Prod. Commun.* 17, 1–7. doi:10.1177/1934578X221105693.
100. Mahmud, S., Afrose, S., Biswas, S., Nagata, A., Paul, G. K., Mita, M. A., et al. (2022). Plant-derived compounds effectively inhibit the main protease of SARS-CoV-2: An in silico approach. *PLoS One* 17, e0273341. doi:10.1371/journal.pone.0273341.
101. Mahmud, S., Biswas, S., Paul, G. K., Mita, M. A., Promi, M. M., Afrose, S., et al. (2021a). Plant-based phytochemical screening by targeting main protease of sars-cov-2 to design effective potent inhibitors. *Biology (Basel).* 10. doi:10.3390/biology10070589.
102. Mahmud, S., Hasan, M. R., Biswas, S., Paul, G. K., Afrose, S., Mita, M. A., et al. (2021b). Screening of Potent Phytochemical Inhibitors Against SARS-CoV-2 Main Protease: An Integrative Computational Approach. *Front. Bioinforma.* 1, 1–15. doi:10.3389/fbinf.2021.717141.
103. Mahmud, S., Mita, M. A., Biswas, S., Paul, G. K., Promi, M. M., Afrose, S., et al. (2021c). Molecular docking and dynamics study to explore phytochemical ligand molecules against the main protease of SARS-CoV-2 from extensive phytochemical datasets. *Expert Rev. Clin. Pharmacol.* 14, 1305–1315. doi:10.1080/17512433.2021.1959318.
104. Mahmud, S., Paul, G. K., Afroze, M., Islam, S., Gupt, S. B. R., Razu, M. H., et al. (2021d). Efficacy of phytochemicals derived from avicennia officinalis for the management of covid-19: A combined in silico and biochemical study. *Molecules* 26. doi:10.3390/molecules26082210.
105. Mahmud, S., Uddin, M. A. R., Paul, G. K., Shimu, M. S. S., Islam, S., Rahman, E., et al. (2021e). Virtual screening and molecular dynamics simulation study of plant-derived compounds to identify potential inhibitors of main protease from SARS-CoV-2. *Brief. Bioinform.* 22, 1402–1414. doi:10.1093/bib/bbaa428.
106. Mahmud, S., Uddin, M. A. R., Zaman, M., Sujon, K. M., Rahman, M. E., Shehab, M. N., et al. (2021f). Molecular docking and dynamics study of natural compound for potential inhibition of main protease of SARS-CoV-2. *J. Biomol. Struct. Dyn.* 39, 6281–6289. doi:10.1080/07391102.2020.1796808.
107. Majeed, A., Hussain, W., Yasmin, F., Akhtar, A., and Rasool, N. (2021). Virtual Screening of Phytochemicals by Targeting HR1 Domain of SARS-CoV-2 S Protein: Molecular Docking, Molecular Dynamics Simulations, and DFT Studies. *Biomed Res. Int.* 2021. doi:10.1155/2021/6661191.
108. Mandal, M. (2021). Phytochemicals as potential inhibitors for novel coronavirus 2019-nCoV/SARS-CoV-2: A graph-based computational analysis. *ACM Int. Conf. Proceeding Ser.* doi:10.1145/3468784.3468886.
109. Manjunathan, R., Periyaswami, V., Mitra, K., Rosita, A. S., Pandya, M., Selvaraj, J., et al. (2022). Molecular docking analysis reveals the functional inhibitory effect of Genistein and Quercetin on TMPRSS2: SARS-COV-2 cell entry facilitator spike protein. *BMC Bioinformatics* 23, 1–15. doi:10.1186/s12859-022-04724-9.
110. Mauriz, E., and Lechuga, L. M. (2021). Current trends in spr biosensing of sars-cov-2 entry inhibitors. *Chemosensors* 9. doi:10.3390/chemosensors9120330.
111. Messaoudi, O., Gouzi, H., El-Hoshoudy, A. N., Benaceur, F., Patel, C., Goswami, D., et al. (2021). Berries anthocyanins as potential SARS-CoV–2 inhibitors targeting the viral attachment and replication; molecular docking simulation. *Egypt. J. Pet.* 30, 33–43. doi:10.1016/j.ejpe.2021.01.001.
112. Milenković, D. A., Dimić, D. S., Avdović, E. H., and Marković, Z. S. (2020). Several coumarin derivatives and their Pd(ii) complexes as potential inhibitors of the main protease of SARS-CoV-2, anin silicoapproach. *RSC Adv.* 10, 35099–35108. doi:10.1039/d0ra07062a.
113. Mir, S. A., Firoz, A., Alaidarous, M., Alshehri, B., Bin Dukhyil, A. A., Banawas, S., et al. (2022). Identification of SARS-CoV-2 RNA-dependent RNA polymerase inhibitors from the major phytochemicals of Nigella sativa: An in silico approach. *Saudi J. Biol. Sci.* 29, 394–401. doi:10.1016/j.sjbs.2021.09.002.
114. Mishra, G. P., Bhadane, R. N., Panigrahi, D., Amawi, H. A., Asbhy, C. R., and Tiwari, A. K. (2021a). The interaction of the bioflavonoids with five SARS-CoV-2 proteins targets: An in silico study. *Comput. Biol. Med.* 134, 104464. doi:10.1016/j.compbiomed.2021.104464.
115. Mishra, G. P., Bhadane, R. N., Panigrahi, D., Amawi, H. A., Asbhy, C. R., and Tiwari, A. K. (2021b). The interaction of the bioflavonoids with five SARS-CoV-2 proteins targets: An in silico study. *Comput. Biol. Med.* 134, 104464. doi:10.1016/j.compbiomed.2021.104464.
116. Mishra, G. P., Bhadane, R. N., Panigrahi, D., Amawi, H. A., Asbhy, C. R., and Tiwari, A. K. (2021c). The interaction of the bioflavonoids with five SARS-CoV-2 proteins targets: An in silico study. *Comput. Biol. Med.* 134, 1–10. doi:10.1016/j.compbiomed.2021.104464.
117. Mohamad, S. A., Zahran, E. M., Fadeel, M. R. A., Albohy, A., and Safwat, M. A. (2021). New acaciin-loaded self-assembled nanofibers as mpro inhibitors against bcv as a surrogate model for sars-cov-2. *Int. J. Nanomedicine* 16, 1789–1804. doi:10.2147/IJN.S298900.
118. Mohapatra, P. K., Chopdar, K. S., Dash, G. C., and Raval, M. K. (2020). In silico Screening of Phytochemicals of Ocimum sanctum against Main Protease of SARS-CoV-2. *ChemRxiv.*

119. Mohideen, A. K. S. (2021). Molecular docking analysis of phytochemical thymoquinone as a therapeutic agent on sars-cov-2 envelope protein. *Biointerface Res. Appl. Chem.* 11, 8389–8401. doi:10.33263/BRIAC111.83898401.
120. Mondal, P., Natesh, J., Abdul Salam, A. A., Thiyagarajan, S., and Meeran, S. M. (2022). Traditional medicinal plants against replication, maturation and transmission targets of SARS-CoV-2: computational investigation. *J. Biomol. Struct. Dyn.* 40, 2715–2732. doi:10.1080/07391102.2020.1842246.
121. Mostafa, I., Mohamed, N. H., Mohamed, B., Almeer, R., Abulmeaty, M. M. A., Bungau, S. G., et al. (2022). In - silico screening of naturally derived phytochemicals against SARS-CoV Main protease. *Environ. Sci. Pollut. Res.* 29, 26775–26791. doi:10.1007/s11356-021-17642-9.
122. Motwalli, O., and Alazmi, M. (2021). Analysis of natural compounds against the activity of SARS-CoV-2 NSP15 protein towards an effective treatment against COVID-19: a theoretical and computational biology approach. *J. Mol. Model.* 27. doi:10.1007/s00894-021-04750-z.
123. Mounika, K., Anupama, B., Pragathi, J., and Gyanakumari, C. (2010). Journal of scientific research. *J. Sci. Res.* 2, 513–524. doi:10.3329/jsr.v2i3.4899.
124. Mpiana, P. T., Ngbolua, K. te N., Tshibangu, D. S. T., Kilembe, J. T., Gbolo, B. Z., Mwanangombo, D. T., et al. (2020). Identification of potential inhibitors of SARS-CoV-2 main protease from Aloe vera compounds: A molecular docking study. *Chem. Phys. Lett.* 754, 137751. doi:10.1016/j.cplett.2020.137751.
125. Muhammad, I. A., Muangchoo, K., Muhammad, A., Ajingi, S., Muhammad, I. Y., Umar, I. D., et al. (2020). A Computational Study to Identify Potential. *Computationtion* 8, 1–14.
126. Muhammad, I., Rahman, N., Gul-E-Nayab, Niaz, S., Basharat, Z., Rastrelli, L., et al. (2021). Screening of potent phytochemical inhibitors against SARS-CoV-2 protease and its two Asian mutants. *Comput. Biol. Med.* 133, 104362. doi:10.1016/j.compbiomed.2021.104362.
127. Mujwar, S., and Harwansh, R. K. (2022). In silico bioprospecting of taraxerol as a main protease inhibitor of SARS-CoV-2 to develop therapy against COVID-19. *Struct. Chem.* 33, 1517–1528. doi:10.1007/s11224-022-01943-x.
128. Mulpuru, V., and Mishra, N. (2021). Computational Identification of SARS-CoV-2 Inhibitor in Tinospora cordifolia, Cinnamomum zeylanicum and Myristica fragrans. *VirusDisease* 32, 511–517. doi:10.1007/s13337-021-00721-3.
129. Mustafa, S., Alomair, L. A., and Hussein, M. (2022). In Silico Analysis Using SARS-CoV-2 Main Protease and a Set of Phytocompounds to Accelerate the Development of Therapeutic Components against COVID-19. *Processes* 10, 1397. doi:10.3390/pr10071397.
130. Muthumanickam, S., Kamaladevi, A., Boomi, P., Gowrishankar, S., and Pandian, S. K. (2021). Indian Ethnomedicinal Phytochemicals as Promising Inhibitors of RNA-Binding Domain of SARS-CoV-2 Nucleocapsid Phosphoprotein: An In Silico Study. *Front. Mol. Biosci.* 8. doi:10.3389/fmolb.2021.637329.
131. Naik, B., Gupta, N., Ojha, R., Singh, S., Prajapati, V. K., and Prusty, D. (2020). High throughput virtual screening reveals SARS-CoV-2 multi-target binding natural compounds to lead instant therapy for COVID-19 treatment. *Int. J. Biol. Macromol.* 160, 1–17. doi:10.1016/j.ijbiomac.2020.05.184.
132. Ngwe Tun, M. M., Toume, K., Luvai, E., Nwe, K. M., Mizukami, S., Hirayama, K., et al. (2022). The discovery of herbal drugs and natural compounds as inhibitors of SARS-CoV-2 infection in vitro. *J. Nat. Med.* 76, 402–409. doi:10.1007/s11418-021-01596-w.
133. Ogidigo, J. O., Iwuchukwu, E. A., Ibeji, C. U., Okpalefe, O., and Soliman, M. E. S. (2022). Natural phyto, compounds as possible noncovalent inhibitors against SARS-CoV2 protease: computational approach. *J. Biomol. Struct. Dyn.* 40, 2284–2301. doi:10.1080/07391102.2020.1837681.
134. Owis, A. I., El-Hawary, M. S., El Amir, D., Refaat, H., Alaaeldin, E., Aly, O. M., et al. (2021). Flavonoids ofSalvadora persicaL. (meswak) and its liposomal formulation as a potential inhibitor of SARS-CoV-2. *RSC Adv.* 11, 13537–13544. doi:10.1039/d1ra00142f.
135. Özdemir, M., Köksoy, B., Ceyhan, D., Sayın, K., Erçağ, E., Bulut, M., et al. (2022). Design and in silico study of the novel coumarin derivatives against SARS-CoV-2 main enzymes. *J. Biomol. Struct. Dyn.* 40, 4905–4920. doi:10.1080/07391102.2020.1863263.
136. Pal, S., Chowdhury, T., Paria, K., Manna, S., Parveen, S., Singh, M., et al. (2022). Brief survey on phytochemicals to prevent COVID-19. *J. Indian Chem. Soc.* 99, 100244. doi:10.1016/j.jics.2021.100244.
137. Panagiotopoulos, A. A., Karakasiliotis, I., Kotzampasi, D. M., Dimitriou, M., Sourvinos, G., Kampa, M., et al. (2021). Natural polyphenols inhibit the dimerization of the sars-cov-2 main protease: The case of fortunellin and its structural analogs. *Molecules* 26. doi:10.3390/molecules26196068.
138. Parida, P. K., Paul, D., and Chakravorty, D. (2020). The natural way forward: Molecular dynamics simulation analysis of phytochemicals from Indian medicinal plants as potential inhibitors of SARS-CoV-2 targets. *Phyther. Res.* 34, 3420–3433. doi:10.1002/ptr.6868.
139. Patel, C. N., Jani, S. P., Jaiswal, D. G., Kumar, S. P., Mangukia, N., Parmar, R. M., et al. (2021). Identification of antiviral phytochemicals as a potential SARS-CoV-2 main protease (Mpro) inhibitor using docking and molecular dynamics simulations. *Sci. Rep.* 11, 1–13. doi:10.1038/s41598-021-99165-4.
140. Peele, K. A., Potla Durthi, C., Srihansa, T., Krupanidhi, S., Ayyagari, V. S., Babu, D. J., et al. (2020). Molecular docking and dynamic simulations for antiviral compounds against SARS-CoV-2: A computational study. *Informatics Med. Unlocked* 19, 100345. doi:10.1016/j.imu.2020.100345.
141. Perera, R. P., Senadheera, S. P. B. M., and Hewapathirana, S. (2022). Computational Study on SARS-CoV-2 Viral Protein Interaction with Natural Compounds of Coriandrum sativum L. *ICARC 2022 - 2nd Int. Conf. Adv. Res. Comput. Towar. a Digit. Empower. Soc.*, 320–325. doi:10.1109/ICARC54489.2022.9753740.
142. Pluskota-Karwatka, D., Hoffmann, M., and Barciszewski, J. (2021). Reducing SARS-CoV-2 pathological protein activity with small molecules. *J. Pharm. Anal.* 11, 383–397. doi:10.1016/j.jpha.2021.03.012.

143. Poochi, S. P., Easwaran, M., Balasubramanian, B., Anbuselvam, M., Meyyazhagan, A., Park, S., et al. (2020). Employing bioactive compounds derived from Ipomoea obscura (L.) to evaluate potential inhibitor for SARS-CoV-2 main protease and ACE2 protein. *Food Front.* 1, 168–179. doi:10.1002/fft2.29.
144. Prasad, S. K., Pradeep, S., Shimavallu, C., Kollur, S. P., Syed, A., Marraiki, N., et al. (2021). Evaluation of Annona muricata Acetogenins as Potential Anti-SARS-CoV-2 Agents Through Computational Approaches. *Front. Chem.* 8, 1–7. doi:10.3389/fchem.2020.624716.
145. Prasanth, D. S. N. B. K., Murahari, M., Chandramohan, V., Bhavya, G., Lakshmana Rao, A., Panda, S. P., et al. (2021a). In-silico strategies of some selected phytoconstituents from Melissa officinalis as SARS CoV-2 main protease and spike protein (COVID-19) inhibitors. *Mol. Simul.* 47, 457–470. doi:10.1080/08927022.2021.1880576.
146. Prasanth, D. S. N. B. K., Murahari, M., Chandramohan, V., Panda, S. P., Atmakuri, L. R., and Guntupalli, C. (2021b). In silico identification of potential inhibitors from Cinnamon against main protease and spike glycoprotein of SARS CoV-2. *J. Biomol. Struct. Dyn.* 39, 4618–4632. doi:10.1080/07391102.2020.1779129.
147. Protein, S.-S., and Strain, U. K. (2021). Dieckol and Its Derivatives as Potential Inhibitors of. 1–17.
148. Puttaswamy, H., Gowtham, H. G., Ojha, M. D., Yadav, A., Choudhir, G., Raguraman, V., et al. (2020). In silico studies evidenced the role of structurally diverse plant secondary metabolites in reducing SARS-CoV-2 pathogenesis. *Sci. Rep.* 10, 1–24. doi:10.1038/s41598-020-77602-0.
149. Qazi, S., Das, S., Khuntia, B. K., Sharma, V., Sharma, S., Sharma, G., et al. (2021). In Silico Molecular Docking and Molecular Dynamic Simulation Analysis of Phytochemicals From Indian Foods as Potential Inhibitors of SARS-CoV-2 RdRp and 3CLpro. *Nat. Prod. Commun.* 16. doi:10.1177/1934578X211031707.
150. Raj, V., Lee, J. H., Shim, J. J., and Lee, J. (2022). Antiviral activities of 4H-chromen-4-one scaffold-containing flavonoids against SARS–CoV–2 using computational and in vitro approaches. *J. Mol. Liq.* 353, 118775. doi:10.1016/j.molliq.2022.118775.
151. Rameshkumar, M. R., Indu, P., Arunagirinathan, N., Venkatadri, B., El-Serehy, H. A., and Ahmad, A. (2021). Computational selection of flavonoid compounds as inhibitors against SARS-CoV-2 main protease, RNA-dependent RNA polymerase and spike proteins: A molecular docking study. *Saudi J. Biol. Sci.* 28, 448–458. doi:10.1016/j.sjbs.2020.10.028.
152. Rolta, R., Salaria, D., Sharma, P. P., Sharma, B., Kumar, V., Rathi, B., et al. (2021). Phytocompounds of Rheum emodi, Thymus serpyllum, and Artemisia annua Inhibit Spike Protein of SARS-CoV-2 Binding to ACE2 Receptor: In Silico Approach. *Curr. Pharmacol. Reports* 7, 135–149. doi:10.1007/s40495-021-00259-4.
153. Rout, J., Swain, B. C., and Tripathy, U. (2022). In silico investigation of spice molecules as potent inhibitor of SARS-CoV-2. *J. Biomol. Struct. Dyn.* 40, 860–874. doi:10.1080/07391102.2020.1819879.
154. Rudrapal, M., Celik, I., Khan, J., Ansari, M. A., Alomary, M. N., Yadav, R., et al. (2022). Identification of bioactive molecules from Triphala (Ayurvedic herbal formulation) as potential inhibitors of SARS-CoV-2 main protease (Mpro) through computational investigations. *J. King Saud Univ. - Sci.* 34, 101826. doi:10.1016/j.jksus.2022.101826.
155. Rudrapal, M., Issahaku, A. R., Agoni, C., Bendale, A. R., Nagar, A., Soliman, M. E. S., et al. (2021). In silico screening of phytopolyphenolics for the identification of bioactive compounds as novel protease inhibitors effective against SARS-CoV-2. *J. Biomol. Struct. Dyn.* 0, 1–17. doi:10.1080/07391102.2021.1944909.
156. Saakre, M., Mathew, D., and Ravisankar, V. (2021). Perspectives on plant flavonoid quercetin-based drugs for novel SARS-CoV-2. *Beni-Suef Univ. J. Basic Appl. Sci.* 10. doi:10.1186/s43088-021-00107-w.
157. Saliu, T. P., Umar, H. I., Ogunsile, O. J., Okpara, M. O., Yanaka, N., and Elekofehinti, O. O. (2021). Molecular docking and pharmacokinetic studies of phytocompounds from Nigerian Medicinal Plants as promising inhibitory agents against SARS-CoV-2 methyltransferase (nsp16). *J. Genet. Eng. Biotechnol.* 19. doi:10.1186/s43141-021-00273-5.
158. Saraswat, J., Singh, P., and Patel, R. (2021). A computational approach for the screening of potential antiviral compounds against SARS-CoV-2 protease: Ionic liquid vs herbal and natural compounds. *J. Mol. Liq.* 326, 115298. doi:10.1016/j.molliq.2021.115298.
159. Sekiou, O., Bouziane, I., Bouslama, Z., and Djemel, A. (2020). In-Silico Identification of Potent Inhibitors of COVID-19 Main Protease (Mpro) and Angiotensin Converting Enzyme 2 (ACE2) from Natural Products: Quercetin, Hispidulin, and Cirsimaritin Exhibited Better Potential Inhibition than Hydroxy-Chloroquine against. *ChemRxiv* 2.
160. Shadrack, D. M., Vuai, S. A. H., Sahini, M. G., and Onoka, I. (2021). In silicostudy of the inhibition of SARS-COV-2 viral cell entry by neem tree extracts. *RSC Adv.* 11, 26524–26533. doi:10.1039/d1ra04197e.
161. Shanmugarajan, D., P., P., Kumar, B. R. P., and Suresh, B. (2020). Curcumin to inhibit binding of spike glycoprotein to ACE2 receptors: Computational modelling, simulations, and ADMET studies to explore curcuminoids against novel SARS-CoV-2 targets. *RSC Adv.* 10, 31385–31399. doi:10.1039/d0ra03167d.
162. Sharbidre, A., Dhage, P., Duggal, H., and Meshram, R. (2021). In silico investigation of tridax procumbens phytoconstituents against sars-cov-2 infection. *Biointerface Res. Appl. Chem.* 11, 12120–12148. doi:10.33263/BRIAC114.1212012148.
163. Sharma, A., Goyal, S., Yadav, A. K., Kumar, P., and Gupta, L. (2022a). In-silico screening of plant-derived antivirals against main protease, 3CLpro and endoribonuclease, NSP15 proteins of SARS-CoV-2. *J. Biomol. Struct. Dyn.* 40, 86–100. doi:10.1080/07391102.2020.1808077.
164. Sharma, A., and Sharma, R. (2022). Computational studies of Some Phytochemicals against COVID 19 through Molecular Docking Approach. 12, 26–30.
165. Sharma, A., Vora, J., Patel, D., Sinha, S., Jha, P. C., and Shrivastava, N. (2022b). Identification of natural inhibitors against prime targets of SARS-CoV-2 using molecular docking, molecular dynamics simulation and MM-PBSA approaches. *J. Biomol. Struct. Dyn.* 40, 3296–3311. doi:10.1080/07391102.2020.1846624.

166. Sharma, J., Kumar Bhardwaj, V., Singh, R., Rajendran, V., Purohit, R., and Kumar, S. (2021). An in-silico evaluation of different bioactive molecules of tea for their inhibition potency against non structural protein-15 of SARS-CoV-2. *Food Chem.* 346, 128933. doi:10.1016/j.foodchem.2020.128933.
167. Shawan, M. M. A. K., Halder, S. K., and Hasan, M. A. (2021). Luteolin and abyssinone II as potential inhibitors of SARS-CoV-2: an in silico molecular modeling approach in battling the COVID-19 outbreak. *Bull. Natl. Res. Cent.* 45. doi:10.1186/s42269-020-00479-6.
168. Singh, R., Bhardwaj, V. K., and Purohit, R. (2021a). Potential of turmeric-derived compounds against RNA-dependent RNA polymerase of SARS-CoV-2: An in-silico approach. *Comput. Biol. Med.* 139, 104965. doi:10.1016/j.compbiomed.2021.104965.
169. Singh, R., Bhardwaj, V. K., Sharma, J., Purohit, R., and Kumar, S. (2022). In-silico evaluation of bioactive compounds from tea as potential SARS-CoV-2 nonstructural protein 16 inhibitors. *J. Tradit. Complement. Med.* 12, 35–43. doi:10.1016/j.jtcme.2021.05.005.
170. Singh, S., Sk, M. F., Sonawane, A., Kar, P., and Sadhukhan, S. (2021b). Plant-derived natural polyphenols as potential antiviral drugs against SARS-CoV-2 via RNA-dependent RNA polymerase (RdRp) inhibition: an in-silico analysis. *J. Biomol. Struct. Dyn.* 39, 6249–6264. doi:10.1080/07391102.2020.1796810.
171. Snoussi, M., Redissi, A., Mosbah, A., De Feo, V., Adnan, M., Aouadi, K., et al. (2021). Emetine, a potent alkaloid for the treatment of SARS-CoV-2 targeting papain-like protease and non-structural proteins: pharmacokinetics, molecular docking and dynamic studies. *J. Biomol. Struct. Dyn.* 0, 1–14. doi:10.1080/07391102.2021.1946715.
172. Srivastava, A. K., Kumar, A., Srivastava, H., and Misra, N. (2022). The role of herbal plants in the inhibition of SARS-CoV-2 main protease: A computational approach. *J. Indian Chem. Soc.* 99, 100640. doi:10.1016/j.jics.2022.100640.
173. Suleimen, Y. M., Jose, R. A., Suleimen, R. N., Arenz, C., Ishmuratova, M., Toppet, S., et al. (2022). Isolation and In Silico Anti-SARS-CoV-2 Papain-like Protease Potentialities of Two Rare 2-Phenoxychromone Derivatives from Artemisia spp. *Molecules* 27, 1–18. doi:10.3390/molecules27041216.
174. Sureja, D. K., Shah, A. P., Gajjar, N. D., Jadeja, S. B., Bodiwala, K. B., and Dhameliya, T. M. (2022). In-silico Computational Investigations of AntiViral Lignan Derivatives as Potent Inhibitors of SARS CoV-2 . *ChemistrySelect* 7. doi:10.1002/slct.202202069.
175. Suručić, R., Radović Selgrad, J., Kundaković-Vasović, T., Lazović, B., Travar, M., Suručić, L., et al. (2022). In Silico and In Vitro Studies of Alchemilla viridiflora Rothm-Polyphenols' Potential for Inhibition of SARS-CoV-2 Internalization. *Molecules* 27. doi:10.3390/molecules27165174.
176. Swain, S. S., Singh, S. R., Sahoo, A., Hussain, T., and Pati, S. (2022). Anti-HIV-drug and phyto-flavonoid combination against SARS-CoV-2: a molecular docking-simulation base assessment. *J. Biomol. Struct. Dyn.* 40, 6463–6476. doi:10.1080/07391102.2021.1885495.
177. Tallei, T. E., Tumilaar, S. G., Lombogia, L. T., Adam, A. A., Sakib, S. A., Emran, T. B., et al. (2021). Potential of betacyanin as inhibitor of SARS-CoV-2 revealed by molecular docking study. *IOP Conf. Ser. Earth Environ. Sci.* 711. doi:10.1088/1755-1315/711/1/012028.
178. Tallei, T. E., Tumilaar, S. G., Niode, N. J., Fatimawali, Kepel, B. J., Idroes, R., et al. (2020). Potential of Plant Bioactive Compounds as SARS-CoV-2 Main Protease (Mpro) and Spike (S) Glycoprotein Inhibitors: A Molecular Docking Study. *Scientifica (Cairo).* 2020. doi:10.1155/2020/6307457.
179. Thakkar, S. S., Shelat, F., and Thakor, P. (2021). Magical bullets from an indigenous Indian medicinal plant Tinospora cordifolia: An in silico approach for the antidote of SARS-CoV-2. *Egypt. J. Pet.* 30, 53–66. doi:10.1016/j.ejpe.2021.02.005.
180. Tietjen, I., Cassel, J., Register, E. T., Zhou, X. Y., Messick, T. E., Keeney, F., et al. (2021). The natural stilbenoid (-)-hopeaphenol inhibits cellular entry of sars-cov-2 usa-wa1/2020, b.1.1.7, and b.1.351 variants. *Antimicrob. Agents Chemother.* 65. doi:10.1128/AAC.00772-21.
181. Tripathi, M. K., Singh, P., Sharma, S., Singh, T. P., Ethayathulla, A. S., and Kaur, P. (2020a). Identification of bioactive molecule from Withania somnifera (Ashwagandha) as SARS-CoV-2 main protease inhibitor. *J. Biomol. Struct. Dyn.* 0, 1–14. doi:10.1080/07391102.2020.1790425.
182. Tripathi, M. K., Singh, P., Sharma, S., Singh, T. P., Ethayathulla, A. S., and Kaur, P. (2020b). Identification of bioactive molecule from Withania somnifera (Ashwagandha) as SARS-CoV-2 main protease inhibitor. *J. Biomol. Struct. Dyn.* 39, 1–14. doi:10.1080/07391102.2020.1790425.
183. Umesh, Kundu, D., Selvaraj, C., Singh, S. K., and Dubey, V. K. (2021). Identification of new anti-nCoV drug chemical compounds from Indian spices exploiting SARS-CoV-2 main protease as target. *J. Biomol. Struct. Dyn.* 39, 3428–3434. doi:10.1080/07391102.2020.1763202.
184. Upreti, S., Prusty, J. S., Pandey, S. C., Kumar, A., and Samant, M. (2021). Identification of novel inhibitors of angiotensin-converting enzyme 2 (ACE-2) receptor from Urtica dioica to combat coronavirus disease 2019 (COVID-19). *Mol. Divers.* 25, 1795–1809. doi:10.1007/s11030-020-10159-2.
185. Vardhan, S., and Sahoo, S. K. (2022). Virtual screening by targeting proteolytic sites of furin and TMPRSS2 to propose potential compounds obstructing the entry of SARS-CoV-2 virus into human host cells. *J. Tradit. Complement. Med.* 12, 6–15. doi:10.1016/j.jtcme.2021.04.001.
186. Verma, S., Patel, C. N., and Chandra, M. (2021). Identification of novel inhibitors of SARS-CoV-2 main protease (Mpro) from Withania sp. by molecular docking and molecular dynamics simulation. *J. Comput. Chem.* 42, 1861–1872. doi:10.1002/jcc.26717.
187. Vijayakumar, M., Janani, B., Kannappan, P., Renganathan, S., Al-Ghamdi, S., Alsaidan, M., et al. (2022). In silico identification of potential inhibitors against main protease of SARS-CoV-2 6LU7 from Andrographis panniculata via molecular docking, binding energy calculations and molecular dynamics simulation studies. *Saudi J. Biol. Sci.* 29, 18–29. doi:10.1016/j.sjbs.2021.10.060.

188. Vincent, S., Arokiyaraj, S., Saravanan, M., and Dhanraj, M. (2020). Molecular Docking Studies on the Anti-viral Effects of Compounds From Kabasura Kudineer on SARS-CoV-2 3CLpro. *Front. Mol. Biosci.* 7, 1–12. doi:10.3389/fmolb.2020.613401.
189. Vivek-Ananth, R.P. .; Abhijit, R.; Nithin, R. . H. S. B. . A. S. I. S. I. of P. N. P., and To, Inhibitors of Human Proteases Key to SARS-CoV-2 infection. Molecules. 2020, 25, 3822. (2020). In Silico Identification of. *Molecules* 25, 3822.
190. Wadanambi, P. M., Jayathilaka, N., and Seneviratne, K. N. (2022). A Computational Study of Carbazole Alkaloids from Murraya koenigii as Potential SARS-CoV-2 Main Protease Inhibitors. *Appl. Biochem. Biotechnol.* doi:10.1007/s12010-022-04138-6.
191. Wang, S.-C., Chen, Y., Wang, Y.-C., Wang, W.-J., Yang, C.-S., Tsai, C.-L., et al. (2020). Tannic acid suppresses SARS-CoV-2 as a dual inhibitor of the viral main protease and the cellular TMPRSS2 protease. *Am. J. Cancer Res.* 10, 4538–4546.
192. Wang, S., Sun, Q., Xu, Y., Pei, J., and Lai, L. (2021). A transferable deep learning approach to fast screen potential antiviral drugs against SARS-CoV-2. *Brief. Bioinform.* 22, 1–11. doi:10.1093/bib/bbab211.
193. Yañez, O., Osorio, M. I., Areche, C., Vasquez-Espinal, A., Bravo, J., Sandoval-Aldana, A., et al. (2021a). Theobroma cacao L. compounds: Theoretical study and molecular modeling as inhibitors of main SARS-CoV-2 protease. *Biomed. Pharmacother.* 140. doi:10.1016/j.biopha.2021.111764.
194. Yañez, O., Osorio, M. I., Areche, C., Vasquez-Espinal, A., Bravo, J., Sandoval-Aldana, A., et al. (2021b). Theobroma cacao L. compounds: Theoretical study and molecular modeling as inhibitors of main SARS-CoV-2 protease. *Biomed. Pharmacother.* 140. doi:10.1016/j.biopha.2021.111764.
195. Yang, L. J., Chen, R. H., Hamdoun, S., Coghi, P., Ng, J. P. L., Zhang, D. W., et al. (2021). Corilagin prevents SARS-CoV-2 infection by targeting RBD-ACE2 binding. *Phytomedicine* 87, 153591. doi:10.1016/j.phymed.2021.153591.
196. Yim, S. K., Kim, I., Warren, B., Kim, J., Jung, K., and Ku, B. (2021). Antiviral activity of two marine carotenoids against sars-cov-2 virus entry in silico and in vitro. *Int. J. Mol. Sci.* 22. doi:10.3390/ijms22126481.
197. Yusuf, A. J., Abdullahi, M. I., Musa, A. M., Abubakar, H., Amali, A. M., and Nasir, A. H. (2022). Potential Inhibitors of SARS-CoV-2 from Neocarya macrophylla (Sabine) Prance ex F. White: Chemoinformatic and Molecular Modeling Studies for Three Key Targets. *Turkish J. Pharm. Sci.* 19, 202–212. doi:10.4274/tjps.galenos.2021.57527.
198. Zhao, Y., Tian, Y., Pan, C., Liang, A., Zhang, W., and Sheng, Y. (2022). Target-Based In Silico Screening for Phytoactive Compounds Targeting SARS-CoV-2. *Interdiscip. Sci. – Comput. Life Sci.* 14, 64–79. doi:10.1007/s12539-021-00461-4.
199. Zothantluanga, J. H., Gogoi, N., Shakya, A., Chetia, D., and Lalthanzara, H. (2021). Computational guided identification of potential leads from Acacia pennata (L.) Willd. as inhibitors for cellular entry and viral replication of SARS-CoV-2. *Futur. J. Pharm. Sci.* 7. doi:10.1186/s43094-021-00348-7.
200. Zrieq, R., Ahmad, I., Snoussi, M., Noumi, E., Iriti, M., Algahtani, F. D., et al. (2021). Tomatidine and patchouli alcohol as inhibitors of SARS-CoV-2 enzymes (3CLpro, PLpro and NSP15) by molecular docking and molecular dynamics simulations. *Int. J. Mol. Sci.* 22. doi:10.3390/ijms221910693.
